# Evaluation of the Therapeutic Effect of Antibiotics on Scrub Typhus: A Systematic Review and Network Meta-Analysis

**DOI:** 10.3389/fpubh.2022.883945

**Published:** 2022-04-27

**Authors:** Dafeng Lu, Taiwu Wang, Zhenghan Luo, Fuqiang Ye, Jiaojiao Qian, Jinhai Zhang, Chunhui Wang

**Affiliations:** ^1^School of Public Health, Nanjing Medical University, Nanjing, China; ^2^Department of Infectious Disease Prevention and Control, Center for Disease Control and Prevention of Eastern Theater, Nanjing, China

**Keywords:** scrub typhus, network meta-analysis, antibiotics, treatment, cure rate

## Abstract

**Background:**

To explore the efficacy and safety of drugs in patients with scrub typhus.

**Methods:**

For this systematic review and network meta-analysis, we searched PubMed, Embase, Web of Science, Cochrane Central Register of Clinical Trials, China National Knowledge Infrastructure (CNKI), and Wanfang data (WF) up to December 2021. All randomized controlled trials (RCTs) of antibiotics used to treat scrub typhus were included without language or date restrictions. The overall effectiveness was evaluated from 4 perspectives: cure rate (CR), defervescence time (DT), gastrointestinal symptoms–adverse events (GS-AD), and abnormal blood count–adverse events (ABC-AD). The quality of evidence was evaluated using the Cochrane Risk of Bias tool and GRADE system.

**Results:**

Sixteen studies involving 1,582 patients were included to evaluate 7 drugs, namely, azithromycin, doxycycline, chloramphenicol, tetracycline, rifampin, moxifloxacin, and telithromycin. In this network meta-analysis, rifampicin (82%) and chloramphenicol (65%) were more effective in terms of CR, and moxifloxacin (3%) from the quinolone family was the worst. Azithromycin caused the fewest events in terms of ABC-AD. No differences were found in this network meta-analysis (NMA) in terms of DT and GS-AD.

**Conclusions:**

Rifampicin was associated with the highest CR benefit and the lowest risk of DT when used to treat patients with scrub typhus, except in areas where tuberculosis (TB) was endemic. Azithromycin was found to be better in CR and was associated with a lower probability of GS-AD and ABC-AD; therefore, it may be considered to treat pregnant women and children. Moxifloxacin had a much lower CR than other drugs and is, therefore, not recommended for the management of scrub typhus.

**Systematic Review Registration:**

https://www.crd.york.ac.uk/PROSPERO/, identifier: CRD42021287837.

## Introduction

Scrub typhus is a leading cause of acute febrile illness in Asia. It is a zoonotic disease caused by the bacterium *Orientia tsutsugamushi* (1). Scrub typhus is endemic in the “Tsutsugamushi Triangle,” which includes Russia in the far east and north, Japan and the Korean Peninsula in the east, northern Australia in the south, and Central Asia in the west (1–3). People infected with scrub typhus show symptoms ranging from mild fever with rash to severe multiorgan dysfunction (4, 5). The incidence of scrub typhus has been increasing significantly in recent years (5, 6), and the number of cases is estimated to exceed 1 million per year (5).

Early access to effective drug therapy is key in reducing disease damage. The commonly used drugs to treat scrub typhus include chloramphenicol, tetracycline, and doxycycline (5). Currently, assessment of the efficacy of drugs used to treat scrub typhus is not well established; thus, in-depth systematic evaluation and guidance are urgently needed.

To provide more evidence in guiding treatment choices for patients with scrub typhus, we conducted a comprehensive systematic review and network meta-analysis of randomized controlled trials (RCTs) to evaluate the efficacy and activity of selected antibiotics.

## Methods

### Search Strategy

We conducted a systematic review and network meta-analysis based on a registered protocol (PROSPERO ID CRD42021287837) and reported it according to the Preferred Reporting Items for Systematic Reviews and Meta-Analyses (PRISMA) guidelines (Supplementary Material S1). Bayesian network meta-analysis was used because it offers a more straightforward method in generating probabilistic statements and in predicting treatment effects.

We searched PubMed, Embase, Cochrane Central Register of Clinical Trials, Web of Science, China National Knowledge Infrastructure (CNKI), and Wan-fang (WF) data, and included reports until December 2021 in all languages. The main search terms used were “Typhus Scrub,” “Antibiotics,” and “Treatment” (Supplementary Material S2). The references of the included articles to articles that were missing from the first search.

### Study Selection

All studies were evaluated and included in the meta-analysis according to the following criteria: (1) Treatment of scrub typhus with antibiotics, including RCTs or quasi-RCTs. (2) Patients were diagnosed with scrub typhus based on clinical symptoms or laboratory tests (7–9).

The exclusion criteria were as follows: (1) RCTs had overlapping patients. (2) The primary outcome was not described or the study reported ambiguous clinical outcomes. (3) The study design was incomplete and/or ambiguous. (4) Combined interventional studies. If several articles were published on the same population intervention, only the study that was complete and of higher quality was included. Two reviewers (DF and ZH) evaluated whether each of the selected RCTs met the intended criteria independently and consulted a third reviewer (TW) in case of a disagreement.

### Data Extraction and Risk of Bias in Network Analysis

Data (e.g., first author, publication year, and patient characteristics), treatments, and reported outcomes were extracted. Extracted data were assessed by two independent authors (DF and ZH) to avoid potential assessment bias.

The risk of bias was assessed for each trial using the Cochrane Handbook for Systematic Reviews of Interventions. Items were considered as low, high, or uncertain risk of bias (10). We incorporated results into the Grading of Recommendations, Assessment, Development, and Evaluation (GRADE) to assess the credibility of findings from each network meta-analysis. The GRADE system evaluates the quality of evidence at four levels: high, moderate, low, and very low (11). Two investigators (DF and TW) independently assessed the risk of bias of individual studies and GRADE system. Any discrepancies were resolved by consensus and arbitration by the authors (DF, JH, TW, and ZH).

### Data Synthesis and Statistical Analysis

We synthesized evidence to compare different treatments in terms of efficacy and safety, and reported our findings as odds ratios (ORs) for binary outcomes [cure rate (CR) and adverse events (ADs)], and as mean differences (MDs) for continuous outcomes [defervescence time (DT)] along with corresponding 95% credible intervals (CrIs). The primary outcome was CR. Secondary outcomes were DT and ADs as reported by the studies.

Network meta-analyses were performed in a Bayesian framework using a Markov chain Monte Carlo simulation technique in gemtc package (R version 4.0.2). For each outcome measure, the fixed-effects and random-effects consistency models were used. Four Markov Chain Monte Carlo (MCMC) were established for running 50,000 iterations, 20,000 sample burn-ins, and a thinning interval of 1. The Brooks-Gelman-Rubin diagnostic was used to evaluate and visualize model convergence of the iterations. Once convergence was established, posterior distributions for model parameters were obtained as the output of the network meta-analysis estimate (MD/OR and the corresponding 95% CrIs). In the presence of minimally informative priors, credible intervals can be interpreted such as conventional confidence intervals (CIs). Network meta-analysis estimated the overall rankings of treatments by calculating the surface under the cumulative ranking curves (SUCRA) for each, which equals 1 when a treatment is the best and 0 when a treatment is the worst. Transitivity was evaluated using descriptive statistics for the study and population baselines, including sample size, age, and gender (12, 13).

We used nodal analysis to evaluate the inconsistency of this NMA by comparing differences between direct and indirect evidence. Heterogeneity between studies was assessed using the I^2^ and P statistic within a visual forest plot. A *p*-value of 0.05 was considered statistically significant. Heterogeneity was considered low, moderate, or high for estimated I^2^ under 25%, between 25 and 50%, and over 50%, respectively. The Egger regression test with a funnel plot was used to assess publication bias, and *p* < 0.05 was considered to indicate significant asymmetry and publication bias. Using the netmeta package in R.

Sensitivity analysis was used to assess the impact of individual or several specific studies on overall heterogeneity. We hypothesized that the inclusion of various study populations and conditions might contribute to heterogeneity and inconsistency. Thus, we assessed the sensitivity of our findings by repeating each network meta-analysis after excluding partial specificity studies (14).

Meta regressions were performed using controlled-placebo adjusted data to examine the relationship between treatment outcome–associated changes and baseline age and sex, publication year and quality of the study, and whether or not it was a randomized study (14, 15). If a multi-arm study was identified based on these meta regressions, the estimates for each group were combined (14). The metafor package in R was used for analysis.

## Results

### Study Selection and Characteristics

**Figure 1:**
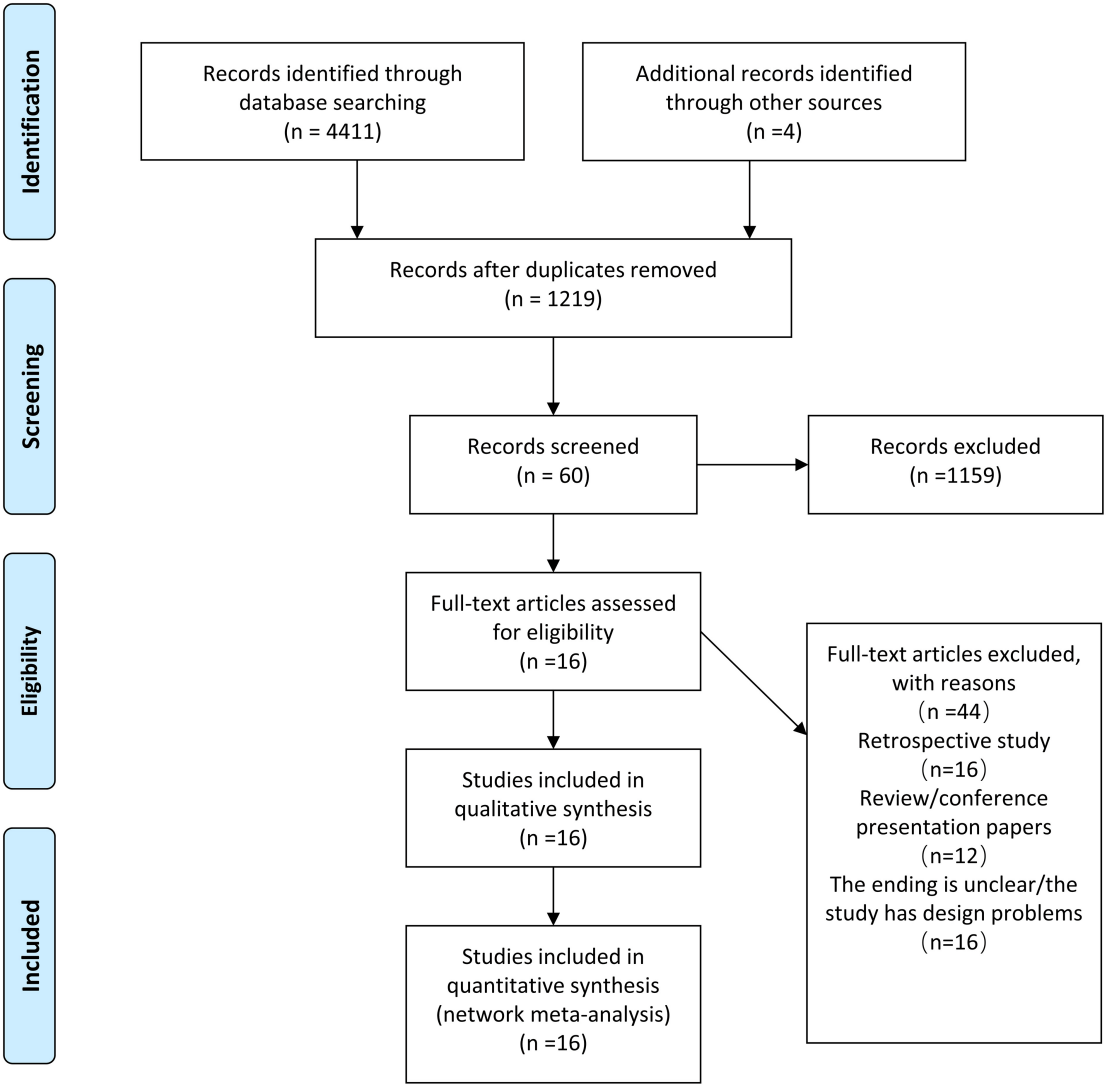
Literature search and selection. The study process followed the PRISMA guidelines.

**Figure 2:**
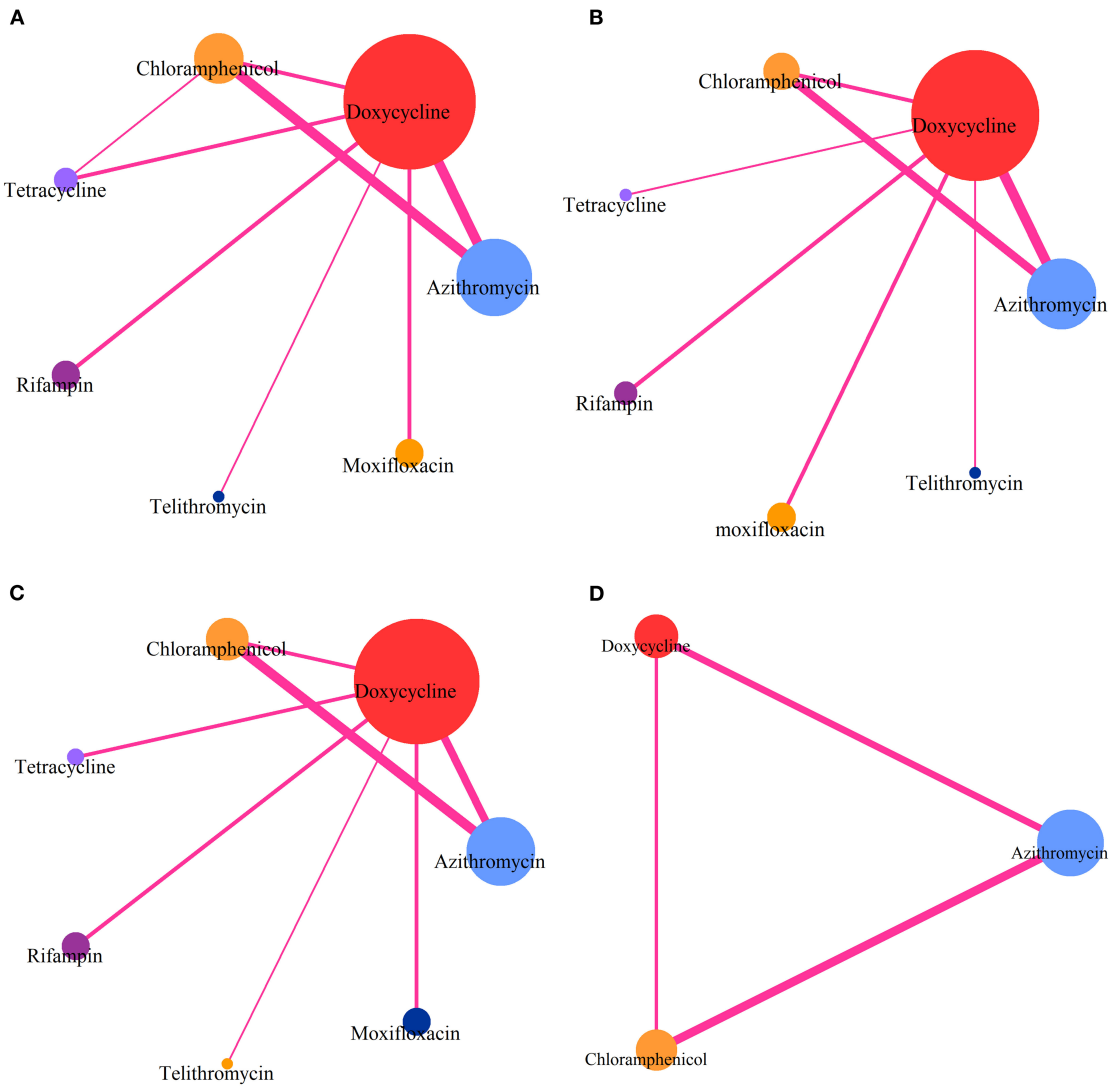
Comparative network plots. **(A)** Comparisons on CR in patients with advanced scrub typhus. **(B)** Comparisons on risk of DT in patients with advanced scrub typhus. **(C)** Comparisons on GS-AD in patients with advanced scrub typhus. **(D)** Comparisons on ABC-AD with advanced scrub typhus. The node size is proportional to the total number of patients receiving a treatment. Each line represents a type of head-to-head comparison. The width of lines is proportional to the number of trials comparing the connected treatments.

The online database search yielded 4,411 results, and 4 were found from a secondary source. After the removal of duplicate entries, a total of 60 articles were further considered for full-text assessment. Finally, 16 studies enrolling a total of 1,582 patients were found to be eligible for further analysis (Figure 1) (7, 16–30). Patients were enrolled to receive any of the following 7 treatments: azithromycin, doxycycline, chloramphenicol, tetracycline, rifampin, moxifloxacin, or telithromycin. Comparative network plots are presented in Figure 2. The main characteristics of included studies are reported in Table 1. The main mechanisms of action of the included drugs are reported in Table 2. The baseline characteristics of studies included in the network meta-analysis have been detailed in Supplementary Materials S3–S5.

**Table 1:** Baseline characteristics of studies included in the network meta-analysis.

**References**	**Study type**	**Year**	**Country**	**Male/female** **(total)**	**Intervention (Experimental: control)**	**Age**	**Diagnosis method**
Chen (16)	RCT	2019	China	49/51 (100)	Doxycycline Moxifloxacin	Dox:51.7 ± 10.1 Mox:51.2 ± 9.7	CD
Kim et al. (17)	Q-RCT	2007	Korea	32/60 (92)	Doxycycline Telithromycin	Dox:61.6 ± 12.8 Tlr:60.2 ± 15.9	CD+IFA/OX-K/IP
Brown et al. (18)	RCT	1978	Malaysia	47/8 (55)	Doxycycline Tetracycline	Dox:30(19-55) Tet:33(18-67)	CD+IFA
Watt et al. (7)	RCT	2000	Thailand	42/36 (78)	Doxycycline Rifampin	Dox:31(19-65) Rfp:33(18-57)	CD+IFA
Song et al. (19)	RCT	1995	Korea	43/73 (116)	Doxycycline Tetracycline	Dox:54.2 ± 13.9 Tet:55 ± 14.8	CD+IFA
Phimda et al. (20)	RCT	2007	Thailand	69.3% (57)	Azithromycin Doxycycline	Azi:38(15-88) Dox:38(15-79)	CD+IFA
Li (21)	RCT	2014	China	27/19 (46)	Azithromycin Chloramphenicol	Azi:32.3 ± 2.5 Chp:34.5 ± 1.6	CD+OX-K
Liang (22)	RCT	2018	China	96/69 (165)	Azithromycin Doxycycline Chloramphenicol	Azi:47.31 ± 0.29 Dox:47.25 ± 0.32/ Chp:47.19 ± 0.33	CD+ IFA/OX-K/IP+MOF
Ruan (22)	Q-RCT	2016	China	60/30 (90)	Azithromycin Doxycycline Chloramphenicol	Azi:46.8 ± 6.5 Dox:47.6 ± 6.8 Chp:47.1 ± 7.0	CD+IFA/OX-K/IP
Sheehy (24)	Q-RCT	1973	Vietnam	NR (60)	Chloramphenicol Tetracycline	Chp: NR Tet: NR	CD+OX-K
Wei et al. (25)	RCT	2004	China	38/29 (67)	Azithromycin Chloramphenicol	6 months−12 years	CD+IFA/OX-K/IP
Wu et al. (26)	RCT	2006	China	74/31 (105)	Azithromycin Chloramphenicol	Azi:86.5 ± 29.7 Chp:85.6 ± 20.3 (month)	CD+OX-K
Kim et al. (27)	RCT	2004	Korea	38/55 (93)	Azithromycin Doxycycline	Azi:62.9 ± 14.1 Dox:61.1 ± 13.5	CD+IFA
Kim et al. (28)	Q-RCT	2018	Korea	59/99 (158)	Doxycycline Rifampin	Dox:63 (52–71) Rfp:67 (52–72)	CD+IFA/PCR
Zhao (29)	Q-RCT	2020	China	90/60 (150)	Azithromycin Doxycycline	Azi:47.33 ± 2.42 Dox:47.26 ± 2.39	CD+IFA/OX-K/IP+MOF
Jie (30)	RCT	2019	China	77/73 (150)	Doxycycline Moxifloxacin	Dox:40.36 ± 3.8 Mox:40.75 ± 4.01	CD+IFA

*RCT, randomized controlled trial; Q-RCT, quasi-randomized controlled trial; Azi, Azithromycin; Dox, Doxycycline; Chp, Chloramphenicol; Tet, Tetracycline; Rfp, Rifampin; Mox, Moxifloxacin; Tlr, Telithromycin; IFA, indirect immunofluorescence antibody test; OX-K, Proteus OX-K agglutinins test; PCR, polymerase chain reaction; IP, isolation of pathogen; CD, Clinical diagnosis; MOF, multiple organ failure; NR, not report*.

**Table 2:** Main mechanisms of action of the included drugs.

**Drug**	**Mechanism of action**
Azithromycin	Azithromycin is a macrolide antibiotic, its mechanism of action is to interfere with protein synthesis by binding to the 50S subunit of the ribosome in bacterial cells and impeding the process of bacterial transpeptide.
Doxycycline	Doxycycline can specifically bind to the 30S subunit of bacterial ribosomes, thus inhibiting aminoacyl-tRNA association at this position, blocking peptide chain extension and interfering with protein synthesis.
Chloramphenicol	Chloramphenicol interferes with the binding of aminoacyl-tRNA terminals to the 50S subunit by reversibly binding to the 50S subunit, thus blocking the formation of new peptide chains and inhibiting protein synthesis. Because the binding of chloramphenicol to the 70S ribosome is reversible, it is considered to be a bacteriostatic antibiotic.
Tetracycline	Tetracycline can inhibit protein synthesis by forming reversible binding to the 30S subunit of bacterial ribosomes. Tetracyclines also inhibit mitochondrial protein synthesis by binding to the mitochondrial 70S subunit.
Rifampin	Rifampin binds to the beta subunit of DNA-dependent RNA polymutase, blocking the enzyme from ligating to DNA and thus blocking the RNA transcription.
Moxifloxacin	Moxifloxacin combines with topoisomerase II and IV to form a triple combination with DNA. It stably disrupts the double-stranded structure of DNA, blocking DNA replication, repair and transcription.
Telithromycin	Telithromycin was produced by the structural modification of macrolides and has high affinity for the binding sites in domains II and V of the 23SrRNA, Inhibiting the synthesis of proteins and blocking their translation and assembly.

### Network Meta-Analysis in Advanced Scrub Typhus

The network meta-analysis included all studies for CR (Figure 2A), 13 studies for DT (Figure 2B), 14 studies for risk of GS-AD (Figure 2C), and 7 studies for risk of ABC-AD (Figure 2D).

**Figure 3:**
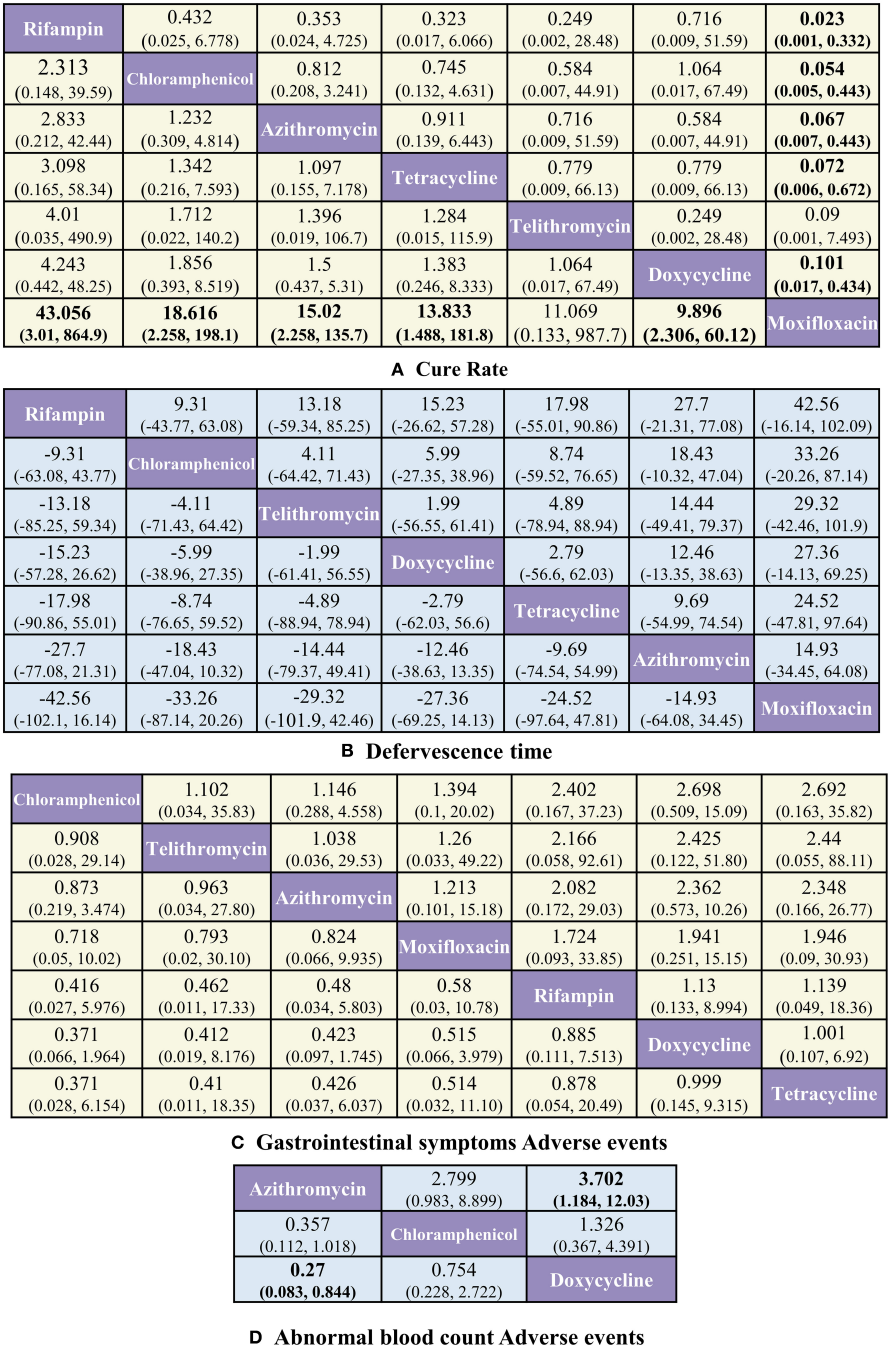
Pooled estimates of the network meta-analysis. **(A)** Pooled odds ratios (95% credible intervals) for CR. **(B)** Pooled Mean difference (95% credible intervals) for DT. **(C)** Pooled odds ratios (95% credible intervals) for GS-AD. **(D)** Pooled odds ratios (95% credible intervals) for ABC-AD. Data in each cell are OR and MD (95% credible intervals) for the comparison of row-defining treatment vs. column-defining treatment. Significant results are highlighted in bold.

With respect to CR (Figure 3A), rifampin was found to have the highest benefit vs. moxifloxacin [OR 43.056, 95%CrI (3.01, 864.9)]. Benefits were also observed with the use of tetracycline [13.83 (1.488, 181.78)], chloramphenicol [18.616 (2.258, 198.05)], azithromycin [15.02 (2.258, 135.7)], and doxycycline [9.896 (2.306, 60.13)] vs. moxifloxacin.

With respect to ABC-AD (Figure 3D), azithromycin was found to have the highest benefit vs. doxycycline [0.27 (0.083, 0.844)], whereas with respect to DT (Figure 3B) and GS-AD (Figure 3C), none of the treatments showed significant differences among each other.

### Rank Probabilities

**Figure 4:**
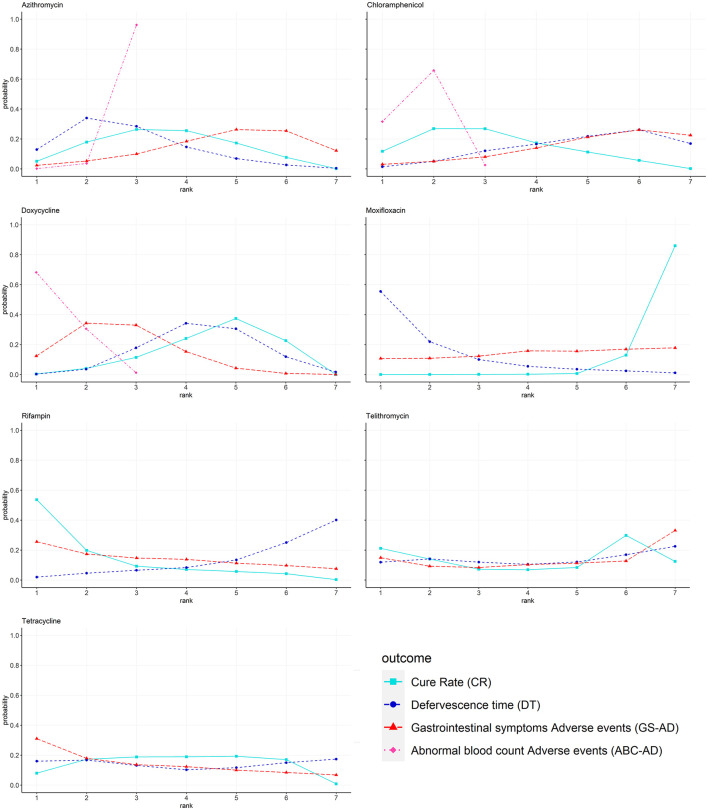
Bayesian ranking profiles of comparable treatments on efficacy for patients with advanced scrub typhus. Profiles indicate the probability of each comparable treatment being ranked from first to last on CR DT GS-AD and ABC-AD. Ranking curves are described according to the Bayesian ranking results presented in Supplementary Material S8.

The Bayesian ranking results were almost in line with those from pooled analyses using ORs and MDs. The meta-analysis revealed that rifampin was most likely to be ranked first for CR (cumulative probability 82%) and DT (23%). Moxifloxacin was most likely to be ranked last for CR (3%) and DT (85%). Doxycycline was most likely to cause GS-AD (72%), followed by tetracycline (66%). Doxycycline had the highest |probability (87%) of being ranked last in causing ABC-AD, followed by chloramphenicol (65%) and azithromycin (2%). The probability profile of each class of drug is shown in Figure 4 and Supplementary Material S8.

### Assessment of Heterogeneity and Inconsistency

The Brooks-Gelman-Rubin diagnostic method demonstrated better stability and replicability for each MCMC chain, with positive confidence in the results (Supplementary Material S12). Results of the evaluation of Inconsistency for all comparisons are presented in Supplementary Material S9. We used a significance level of *P* > 0.05 for all cases. This finding indicates positive transitivity and consistency among comparisons. Heterogeneity was assessed using the I^2^ and P statistic statistics. Among the included trials, the heterogeneity in the CR, AD, and DT groups was determined to be as follows: CR: I^2^ = 1.28%, ABC-AD: I^2^ = 0%, GS-AD: I^2^ = 60.48%, DT: I^2^ = 95.7%. Egger regression tests were performed with *P*-values of CR: 0.14, ABC-AD: 0.63, GS-AD: 0.52, and DT: 0.41, as shown in Supplementary Materials S10, S11.

### Assessment of Risk of Bias and Grade

Overall, in combination with previous studies risk of bias assessments, these trials were considered moderate-high risk for bias. Supplementary Material S7 summarizes the detailed risk of bias assessments. The certainty of evidence was evaluated using the GRADE system (Supplementary Material S13). A small number were rated as “Moderate” (4/21 in CR and 3/3 in ABC-AD), the others were rated as “Low” or “Very Low”.

### Sensitivity Analysis and Meta Regression Analysis

The sensitivity analysis for 3 outcomes (sensitivity analysis and regression analysis were not performed for the ABC-AD group because of the small number of studies) were assessed by repeating analyses after the sequential exclusion of the following studies: 5 studies that did not use randomization, 3 studies that were published prior to 2000, 2 studies of scrub typhus in adolescents, 3 studies that had fewer than 30 patients per arm, and 7 studies that included intravenous interventions. The inclusion of these studies did not have a significant impact on the results. The assessment of heterogeneity (I^2^) was similar across studies, except that I^2^ was reduced noticeably in the paired meta-analysis and network meta-analysis when publications prior to 2000 or a group of fewer than 30 patients were excluded in the GS-AD group (Supplementary Material S14).

In meta regressions analysis, greater drug therapy–induced GS-ADs were associated with higher proportion of male rate [Coefficient = 0.42 percentage^−1^ (0.004–0.081), z = 2.13, *p* = 0.033] and higher mean age of the study [Coefficient = 0.03 age^−1^ (0.003–0.057), z = 2.17, *p* = 0.0301]. We did not find evidence that the incidence of GS-AD was related to study blinding, or paper publication year. The increase in DT was associated with study blinding [Coefficient = 8.71 (4.80–12.62), z = 4.368, *p* ≤ 0.001] and closer paper publication year [Coefficient = −0.34 year^−1^ (−0.62 to −0.06), z = −2.35, *p* = 0.018]. We did not find evidence that DT was related to the proportion of male patients or the mean age of patients in the study. Moreover, we did not find evidence that CR was related to these baseline differences (Supplementary Material S15).

## Discussion

Of the 4415 citations that were retrieved, 16 studies examining the following drugs met the inclusion criteria: azithromycin, doxycycline, chloramphenicol, tetracycline, rifampin, telithromycin, and moxifloxacin. These studies were from Thailand, Vietnam, China (mainly eastern and southern China), Malaysia, Korea, namely, from the traditional “Tsutsugamushi Triangle.” In the CR group, the effect of rifampicin was greater among the drugs, whereas that of moxifloxacin was worse. In terms of toxicity, doxycycline had the highest rate of GS-AD and ABC-AD, whereas azithromycin had a low incidence of adverse reactions for both of these aspects. In the DT group, there were no significant differences between drugs. We found that a higher proportion of males and those with a higher mean age in the study showed a higher incidence of GS-AD, suggesting that males and elderly patients are more sensitive to drug toxicity and that the treatment of this group requires more attention. We found that study blinding and using older papers (those with earlier publication years) increased the extended DT, suggesting that open-label study may produce some reporting bias as well as improved quality of medical care and supportive treatment that reduced the duration of disease. However, there may have been confounding factors that were not identified (e.g., age or gender, which led to differences in the duration of illness before admission and the extent of disease severity). These factors were reported to be substantially missing, making systematic analysis difficult. Our meta-regression analyses were based on study-level data; therefore, we were unable to examine the influence of various factors on the outcomes in detail.

Some studies have noted that chloramphenicol can cause more severe rates of ABC-ADs, including leukopenia, neutropenia, and even aplastic anemia. However, some studies have also concluded that the short-term use of chloramphenicol does not cause serious ABC-ADs (31–33). In this NMA, drug-induced abnormalities of blood indicators were also reported. We found that Doxycycline and Chloramphenicol had a higher incidence of ABC-AD than Azithromycin, with Doxycycline's being statistically significant, which is a phenomenon that needs caution. However, RCTs were generally quite short. We could not analyze them due to a lack of follow-up for drug-generated toxicity.

Doxycycline, the most widely used antibiotic, has the advantage of better CR and shorter DT, but his higher GS-AD and ABC-AD are not negligible problems. When using doxycycline, it is important to choose its dose and duration of treatment carefully. Azithromycin is an FDA-approved drug for use in pregnant women and children (34–36). It has a better CR and a lower probability of AD. Therefore, azithromycin may be considered in the treatment of pregnant women and children. Moxifloxacin had a considerably lower CR than other drugs examined in this study; therefore, it is not recommended for treatment.

Rifampicin is associated with the highest CR benefit and lowest risk of DT benefits for patients as recommended drugs for scrub typhus. Although only 2 studies of rifampicin were included, the robustness of the results was confirmed by a low between-trial heterogeneity, undetected of inconsistency, and a good model fit in the CR trial, and further confirmed by sensitivity analysis and meta-regression. However, rifampicin use may lead to increased drug resistance during the treatment of tuberculosis (9, 34). Therefore, in TB-endemic areas, rifampicin is not recommended as the first choice of drug to treat scrub typhus. In areas with a high incidence of TB, we recommend azithromycin or doxycycline as the recommended drug for patients because of their better therapeutic effect. Azithromycin is more suitable for patients with suitable financial conditions, and doxycycline on the contrary (20).

In some studies with chloramphenicol and doxycycline, there were a small number of disease relapses (7, 17, 19, 24). Some studies have analyzed this phenomenon as being related to the rapid metabolism of the drugs and their short half-life in the blood. Therefore, when using these two drugs, it is recommended to maintain the drug concentration in the blood for a period of time to prevent relapse by still giving the drug for a few days after the symptoms of the disease have disappeared. Some azithromycin (6/8) and chloramphenicol (5/6) treatments used intravenous administration, which may improve the rate and efficiency of drug absorption, lower GS-ADs, and increase the possibility of other ADs. This may partially explain the lower gastrointestinal side effects and higher blood side effects of chloramphenicol.

The use of antibiotics to treat scrub typhus is also associated with bacterial resistance (37, 38). Some studies have isolated strains that are drug-resistant to some antibiotics (e.g., doxycycline, moxifloxacin) (7, 30), but there is a lack of studies have reported the distribution of antibiotic resistance in scrub typhus (39). We attempted to use environmental mean antibiotic contamination in an area to predict local scrub typhus resistance. We reviewed studies that evaluated antibiotic contamination in soil and groundwater. High levels of sulfonamide, chloramphenicol, tetracycline, and quinolone antibiotics were reported, and contamination was found to be widespread in multiple locations in China (40). These findings indicate a situation of antibiotic abuse, which may lead to increased antibiotic resistance and reduces drug efficacy.

Our findings were not completely aligned with those from a recently published network meta-analysis (41). Evidence from their prospective study was inadequate to recommend any of the drugs to treat scrub typhus. Retrospective studies have shown that clarithromycin has a more effective DT. The discrepancy between our results and those of some previously documented results may be due to the large sample sizes used in our study and the restriction of our analyses to RCTs focusing on the treatment of acute conditions.

Our study has several limitations. First, our network meta-analysis included only one study containing telithromycin, which means that the representation and robustness of telithromycin evidence is poor. Second, only a short-term study was performed to evaluate the drug efficacy in treating scrub typhus; therefore, only short-term adverse drug reactions were recorded. Thus, it is difficult to assess the chronic effects of the drug. Third, there were differences in the diagnostic methods used to analyze treatment outcomes related to scrub typhus. Some studies show that the Weil-Felix test is less accurate than the indirect immunofluorescence test and could lead to potential heterogeneity (42, 43). Fourth, determination of AD and DT may have differed between investigators, and some symptoms may have been inadvertently missed, likely explaining the heterogeneity between the GS-AD and DT groups. Thus, an in-depth analysis was a challenge we faced in this study.

## Conclusions

Rifampicin was associated with the highest CR benefit and the lowest risk of DT benefits for patients when recommended to treat scrub typhus, except in areas where TB was endemic. Azithromycin had a better in CR and lower probability of GS-AD and ABC-AD. Therefore, azithromycin may be considered in the treatment of pregnant women and children. Moxifloxacin had a considerably lower CR than the other drugs, and is, therefore, not suggested to treat scrub typhus.

## Data Availability Statement

The original contributions presented in the study are included in the article/Supplementary Material, further inquiries can be directed to the corresponding author/s.

## Author Contributions

CW, JZ, and DL contributed to conception and design of the study. TW organized the database. DL performed the statistical analysis and wrote the first draft of the manuscript. FY, ZL, and TW wrote sections of the manuscript. All authors contributed to manuscript revision, read, and approved the submitted version.

## Funding

This work was sponsored by Military Logistics Scientific Research Program(Nos. BLB19J017 and 19SWAQ19).

## Conflict of Interest

The authors declare that the research was conducted in the absence of any commercial or financial relationships that could be construed as a potential conflict of interest.

## Publisher's Note

All claims expressed in this article are solely those of the authors and do not necessarily represent those of their affiliated organizations, or those of the publisher, the editors and the reviewers. Any product that may be evaluated in this article, or claim that may be made by its manufacturer, is not guaranteed or endorsed by the publisher.
