# Supplementary material for: Evaluation of the Therapeutic Effect of Antibiotics on Scrub Typhus: A Systematic Review and Network Meta-Analysis
Source: Front Public Health. 2022 Apr 27;10:883945. doi: 10.3389/fpubh.2022.883945 (PMC9092376; doi:10.3389/fpubh.2022.883945)
Supplement: Supplementary file 1 [file Data_Sheet_1.docx]

**Supplementary Materials S1:** PRISMA checklist for meta-analysis.

**Supplementary Materials S2:** Search strategy

**Supplementary Materials S3:** Detailed outcome of studies included in the network meta-analysis.

**Supplementary Materials S4:** Detailed interventions of studies included in the network meta-analysis.

**Supplementary Materials S5:** Specific DT outcome of studies included in the network meta-analysis.

**Supplementary Materials S6:** Conversion formula

**Supplementary Materials S7:** Risk of bias analysis detailed for each single study included in the NMA.

(A) Risk-of-bias graph: Each risk-of-bias item presented as percentages across all included studies. (B) Risk-of-bias summary: Each risk-of-bias item for each included study.

**Supplementary Materials S8:** Bayesian ranking results

**Supplementary Materials S9:** Inconsistency of the network meta-analysis.

(A) Pooled odds ratios (95% credible intervals) for CR. (B) Pooled Mean difference (95% credible intervals) for DT. (C) Pooled odds ratios (95% credible intervals) for GS-AE. (D) Pooled odds ratios (95% credible intervals) for ABC-AE.

**Supplementary Materials S10:** Heterogeneity of the network meta-analysis.

(A) Pooled odds ratios (95% credible intervals) for CR. (B) Pooled Mean difference (95% credible intervals) for DT. (C) Pooled odds ratios (95% credible intervals) for GS-AE. (D) Pooled odds ratios (95% credible intervals) for ABC-AE.

**Supplementary Materials S11:** The Funnel plots and asymmetry linear regression test of the network meta-analysis.

(A) Funnel plots and P-value for CR. (B) Funnel plots and P-value for DT. (C) Funnel plots and P-value for GS-AE. (D) Funnel plots and P-value for ABC-AE.

**Supplementary Materials S12:** Brooks-Gelman-Rubin history and diagnostic of the network meta-analysis.

(A) Brooks-Gelman-Rubin history and diagnostic plot for CR. (B) Brooks-Gelman-Rubin history and diagnostic plot for DT. (C) Brooks-Gelman-Rubin history and diagnostic plot for GS-AE. (D) Brooks-Gelman-Rubin history and diagnostic plot for ABC-AE.

**Supplementary Materials S13:** GRADE ratings for 4 network meta-analysis.

**Supplementary Materials S14:** Sensitivity analysis of the network meta-analysis.

Excluded A: exclusion of studies examining did not use randomize, Excluded B: exclusion of studies publications prior to 2000, Excluded C: exclusion of studies with adolescent scrub typhus, Excluded D:exclusion of studies less than 30 patients per arm, Excluded E:exclusion of studies use intravenous.

**Supplementary Materials S15:** meta regressions analysis of the network meta-analysis.

**Supplementary Materials S1**

PRISMA checklist for meta-analysis

| **Section and Topic** | **Item #** | **Checklist item** | **Location where item is reported** |
| --- | --- | --- | --- |
| **TITLE** | | |  |
| Title | 1 | Identify the report as a systematic review. | 1 |
| **ABSTRACT** | | |  |
| Abstract | 2 | See the PRISMA 2020 for Abstracts checklist. | 3 |
| **INTRODUCTION** | | |  |
| Rationale | 3 | Describe the rationale for the review in the context of existing knowledge. | 2 |
| Objectives | 4 | Provide an explicit statement of the objective(s) or question(s) the review addresses. | 2 |
| **METHODS** | | |  |
| Eligibility criteria | 5 | Specify the inclusion and exclusion criteria for the review and how studies were grouped for the syntheses. | 3 |
| Information sources | 6 | Specify all databases, registers, websites, organisations, reference lists and other sources searched or consulted to identify studies. Specify the date when each source was last searched or consulted. | 3 |
| Search strategy | 7 | Present the full search strategies for all databases, registers and websites, including any filters and limits used. | 3 |
| Selection process | 8 | Specify the methods used to decide whether a study met the inclusion criteria of the review, including how many reviewers screened each record and each report retrieved, whether they worked independently, and if applicable, details of automation tools used in the process. | 3 |
| Data collection process | 9 | Specify the methods used to collect data from reports, including how many reviewers collected data from each report, whether they worked independently, any processes for obtaining or confirming data from study investigators, and if applicable, details of automation tools used in the process. | 3 |
| Data items | 10a | List and define all outcomes for which data were sought. Specify whether all results that were compatible with each outcome domain in each study were sought (e.g. for all measures, time points, analyses), and if not, the methods used to decide which results to collect. | 3 |
|  | 10b | List and define all other variables for which data were sought (e.g. participant and intervention characteristics, funding sources). Describe any assumptions made about any missing or unclear information. | 3 |
| Study risk of bias assessment | 11 | Specify the methods used to assess risk of bias in the included studies, including details of the tool(s) used, how many reviewers assessed each study and whether they worked independently, and if applicable, details of automation tools used in the process. | 3 |
| Effect measures | 12 | Specify for each outcome the effect measure(s) (e.g. risk ratio, mean difference) used in the synthesis or presentation of results. | 3，4 |
| Synthesis methods | 13a | Describe the processes used to decide which studies were eligible for each synthesis (e.g. tabulating the study intervention characteristics and comparing against the planned groups for each synthesis (item #5)). | 3 |
|  | 13b | Describe any methods required to prepare the data for presentation or synthesis, such as handling of missing summary statistics, or data conversions. | 3  Supplementary Materials S4-2 |
|  | 13c | Describe any methods used to tabulate or visually display results of individual studies and syntheses. | 3 |
|  | 13d | Describe any methods used to synthesize results and provide a rationale for the choice(s). If meta-analysis was performed, describe the model(s), method(s) to identify the presence and extent of statistical heterogeneity, and software package(s) used. | 4 |
|  | 13e | Describe any methods used to explore possible causes of heterogeneity among study results (e.g. subgroup analysis, meta-regression). | 4 |
|  | 13f | Describe any sensitivity analyses conducted to assess robustness of the synthesized results. | 4 |
| Reporting bias assessment | 14 | Describe any methods used to assess risk of bias due to missing results in a synthesis (arising from reporting biases). | 3 |
| Certainty assessment | 15 | Describe any methods used to assess certainty (or confidence) in the body of evidence for an outcome. | 3 |
| **RESULTS** | | |  |
| Study selection | 16a | Describe the results of the search and selection process, from the number of records identified in the search to the number of studies included in the review, ideally using a flow diagram. | Figure1 |
|  | 16b | Cite studies that might appear to meet the inclusion criteria, but which were excluded, and explain why they were excluded. | NA |
| Study characteristics | 17 | Cite each included study and present its characteristics. | Table1  Supplementary Materials S3  Supplementary Materials S4 |
| Risk of bias in studies | 18 | Present assessments of risk of bias for each included study. | Supplementary Materials S7 |
| Results of individual studies | 19 | For all outcomes, present, for each study: (a) summary statistics for each group (where appropriate) and (b) an effect estimate and its precision (e.g. confidence/credible interval), ideally using structured tables or plots. | Supplementary MaterialsS2,  Supplementary MaterialsS5 |
| Results of syntheses | 20a | For each synthesis, briefly summarise the characteristics and risk of bias among contributing studies. | 4 |
|  | 20b | Present results of all statistical syntheses conducted. If meta-analysis was done, present for each the summary estimate and its precision (e.g. confidence/credible interval) and measures of statistical heterogeneity. If comparing groups, describe the direction of the effect. | Figure 3,4,5  Supplementary Materials S7 |
|  | 20c | Present results of all investigations of possible causes of heterogeneity among study results. | 5 |
|  | 20d | Present results of all sensitivity analyses conducted to assess the robustness of the synthesized results. | 5 |
| Reporting biases | 21 | Present assessments of risk of bias due to missing results (arising from reporting biases) for each synthesis assessed. | 5 |
| Certainty of evidence | 22 | Present assessments of certainty (or confidence) in the body of evidence for each outcome assessed. | 5 |
| **DISCUSSION** | | |  |
| Discussion | 23a | Provide a general interpretation of the results in the context of other evidence. | 6, 7 |
|  | 23b | Discuss any limitations of the evidence included in the review. | 7 |
|  | 23c | Discuss any limitations of the review processes used. | 7 |
|  | 23d | Discuss implications of the results for practice, policy, and future research. | 6, 7 |
| **OTHER INFORMATION** | | |  |
| Registration and protocol | 24a | Provide registration information for the review, including register name and registration number, or state that the review was not registered. | 1, 3 |
|  | 24b | Indicate where the review protocol can be accessed, or state that a protocol was not prepared. | 1, 3 |
|  | 24c | Describe and explain any amendments to information provided at registration or in the protocol. | 1, 3 |
| Support | 25 | Describe sources of financial or non-financial support for the review, and the role of the funders or sponsors in the review. | Appendix |
| Competing interests | 26 | Declare any competing interests of review authors. | No competing |
| Availability of data, code and other materials | 27 | Report which of the following are publicly available and where they can be found: template data collection forms; data extracted from included studies; data used for all analyses; analytic code; any other materials used in the review. | Appendix |

**Supplementary Materials S2**

**Search strategy**

Search date: December 6, 2021

**We search EMBASE (**1,962 records were found**), Pubmed (**629 records were found**) using the following terms:**

**1**

(Tsutsugamushi Fever or Tsutsugamushi Fevers or (Fever and Tsutsugamushi) or (Fevers and Tsutsugamushi) or Tsutsugamushi Disease or Tsutsugamushi Diseases or (Disease and Tsutsugamushi) or (Diseases and Tsutsugamushi) or Orientia tsutsugamushi Infection or Orientia tsutsugamushi Infections or (Infection and Orientia tsutsugamushi) or (Infections and Orientia tsutsugamushi) or Scrub Typhus or ( Scrub and Typhus)) .mp.

[mp=ti, ab, hw, tn, ot, dm, mf, dv, kw, fx, dq, nm, kf, ox, px, rx, ui, sy, tc, id, tm]

**2**

(Therapeutic or Therapy or Therapies or Treatment or Treatments or Drug or Drugs) .mp.

**3**

(Antibacterial Agent or Anti-Bacterial Agent or Bacteriocide or Bacteriocides or Antibiotic or Antibiotics) .mp.

**4**

2 or 3

**5**

1 and 4

**We search Cochrane Central Register of Clinical Trials (**34 records were found**), Web of Science (**1529 records were found**) using the following terms:**

(Tsutsugamushi Fever OR Tsutsugamushi Fevers OR (Fever AND Tsutsugamushi) OR (Fevers AND Tsutsugamushi) OR Tsutsugamushi Disease OR Tsutsugamushi Diseases OR (Disease AND Tsutsugamushi) OR (Diseases AND Tsutsugamushi) OR Orientia tsutsugamushi Infection OR Orientia tsutsugamushi Infections OR (Infection and Orientia tsutsugamushi) OR (Infections and Orientia tsutsugamushi) OR Scrub Typhus OR ( Scrub AND Typhus)) AND ((Therapeutic OR Therapy OR Therapies OR Treatment OR Treatments OR Drug or Drugs) OR (Antibacterial Agent OR Anti-Bacterial Agent OR Bacteriocide OR Bacteriocides OR Antibiotic OR Antibiotics))

**We searched China National Knowledge Infrastructure (**168 records were found**) and Wan-fang data (**89 records were found**) using the keywords "tsutsugamushi", "treatment", "antibiotics" and their extensions in Chinese, and performed a preliminary screening on the search.**

**Supplementary Materials S3**

Detailed outcome of studies included in the network meta-analysis.

| study | drug | n | Report Outcome | | | | | | | | | Adverse events | | | | | | | | | | | |
| --- | --- | --- | --- | --- | --- | --- | --- | --- | --- | --- | --- | --- | --- | --- | --- | --- | --- | --- | --- | --- | --- | --- | --- |
|  |  |  | primary | | Other (Symptom resolution time) | | | | | | | Gastrointestinal symptoms | | | | | Abnormal blood count | | | | Rae | Diz | EC |
|  |  |  | C/F | DT | Hda | Myg | Esh | Ras | Cou | LOS | Lnp | Vom | Dia | Nau | Abd | Gas | Glp | Thp | Lkp | ALT |  |  |  |
| Chen 2019 | Dox | 50 | 49  23 | √ |  |  |  |  |  |  |  | 2 | 2 |  | 1 |  |  |  |  |  |  | 1 |  |
|  | Mox | 50 |  |  |  |  |  |  |  |  |  | 3 | 2 |  | 0 |  |  |  |  |  |  | 1 |  |
| Kim 2007 | Dox | 45 | 44  47 | √ | √ | √ |  | √ |  |  |  | 1 | 1 | 2 | 2 |  |  |  |  | 2 | 2 |  | 1 |
|  | Tlr | 47 |  |  |  |  |  |  |  |  |  | 0 | 0 | 0 | 3 |  |  |  |  | 4 | 0 |  | 0 |
| Brown 1978 | Dox | 31 | 31  24 |  |  |  |  |  |  |  |  | 8 |  |  |  |  |  |  |  |  | 1 |  |  |
|  | Tet | 24 |  |  |  |  |  |  |  |  |  | 0 |  |  |  |  |  |  |  |  | 0 |  |  |
| Watt 2000 | Dox | 28 | 24  50 | √ | √ | √ |  |  | √ |  |  |  |  |  |  | 14 |  |  |  |  | 1 |  |  |
|  | Rfp | 50 |  |  |  |  |  |  |  |  |  |  |  |  |  | 18 |  |  |  |  | 7 |  |  |
| Song 1995 | Dox | 66 | 62  50 | √ | √ | √ |  |  |  |  |  |  |  |  |  | 13 |  |  |  |  |  |  |  |
|  | Tet | 50 |  |  |  |  |  |  |  |  |  |  |  |  |  | 25 |  |  |  |  |  |  |  |
| Phimda2007 | Azi | 30 | 29  27 | √ |  |  |  |  |  |  |  | not reported | | |  |  |  |  |  |  |  |  |  |
|  | Dox | 27 |  |  |  |  |  |  |  |  |  |  |  |  |  |  |  |  |  |  |  |  |  |
| Li 2014 | Azi | 23 | 23  23 | √ |  |  | √ |  |  |  | √ |  |  |  |  | 2 | 0 |  |  |  |  |  |  |
|  | Chp | 23 |  |  |  |  |  |  |  |  |  |  |  |  |  | 4 | 4 |  |  |  |  |  |  |
| Liang 2018 | Azi | 55 | 55  55  55 | √ |  |  | √ |  |  | √ |  |  |  | 1 | 1 |  | 0 |  | 0 | 0 |  |  |  |
|  | Dox | 55 |  |  |  |  |  |  |  |  |  |  |  | 2 | 3 |  | 3 |  | 3 | 3 |  |  |  |
|  | Chp | 55 |  |  |  |  |  |  |  |  |  |  |  | 2 | 2 |  | 1 |  | 2 | 1 |  |  |  |
| Ruan 2016 | Azi | 30 | 30  30  30 | √ |  |  | √ |  | √ |  | √ |  |  | 1 | 0 |  | 0 |  |  |  |  |  |  |
|  | Dox | 30 |  |  |  |  |  |  |  |  |  |  |  | 3 | 2 |  | 0 |  |  |  |  |  |  |
|  | Chp | 30 |  |  |  |  |  |  |  |  |  |  |  | 2 | 2 |  | 2 |  |  |  |  |  |  |
| Sheehy 1973 | Chp | 30 | 25  22 | √ |  |  |  |  |  | √ |  | not reported | | |  |  |  |  |  |  |  |  |  |
|  | Tet | 30 |  |  |  |  |  |  |  |  |  |  |  |  |  |  |  |  |  |  |  |  |  |
| Wei 2004 | Azi | 34 | 31  31 |  |  |  |  |  |  |  |  |  |  |  | 4 |  |  |  | 0 |  |  |  |  |
|  | Chp | 33 |  |  |  |  |  |  |  |  |  |  |  |  | 0 |  |  |  | 1 |  |  |  |  |
| Wu 2006 | Azi | 52 | 52  53 | √ |  |  | √ |  |  |  |  |  |  |  | 9 |  |  |  | 0 |  |  |  |  |
|  | Chp | 53 |  |  |  |  |  |  |  |  |  |  |  |  | 0 |  |  |  | 3 |  |  |  |  |
| Kim 2004 | Azi | 47 | 47  43 | √ |  |  |  |  |  |  |  | 0 | 2 | 4 | 1 |  |  | 1 |  | 5 |  |  |  |
|  | Dox | 46 |  |  |  |  |  |  |  |  |  | 3 | 0 | 6 | 3 |  |  | 0 |  | 4 |  |  |  |
| Kim 2018 | Dox | 83 | 83  75 | √ | √ | √ |  | √ |  |  |  |  | 2 |  |  |  |  |  |  | 5 | 3 |  | 4 |
|  | Rfp | 75 |  |  |  |  |  |  |  |  |  | 1 | 1 |  | 1 |  |  |  |  | 6 | 1 |  | 4 |
| Zhao 2020 | Azi | 75 | 70  69 | √ |  |  | √ |  |  | √ |  |  |  | 1 | 1 |  | 1 |  | 1 | 1 |  |  |  |
|  | Dox | 75 |  |  |  |  |  |  |  |  |  |  |  | 4 | 3 |  | 3 |  | 3 | 2 |  |  |  |
| Jie 2019 | Dox | 75 | 67  55 | √ | √ |  | √ |  |  |  |  |  | 3 | 6 | 3 |  |  |  |  |  | 2 | 1 |  |
|  | Mox | 75 |  |  |  |  |  |  |  |  |  |  | 1 | 3 | 0 |  |  |  |  |  | 1 | 0 |  |

Azi, Azithromycin; Dox, Doxycycline; Chp, Chloramphenicol; Tet, Tetracycline; Rfp, Rifampin; Mox, Moxifloxacin; Tlr, Telithromycin; C/F, Cure rate; DT, Defervescence time; Vom, Vomiting; Dia, Diarrhea; Nau, Nausea; Abd, Abdominal discomfort; Gas, Gastrointestinal only; Glp, Granulocytopenia; Thp, Thrombocytopenia; Lkp, Leukopenia; ALT, Elevated serum ALT level; Rae, Rash emergence; Diz, Dizziness; EC, Esophageal candidiasis; n, participant; Had, Headache, Myg, Myalgia; Esh, Eschar; Ras, Rash subsides; Cou, Cough; LOS, Length Of Stay; Lnp, Lymph node pain

**Supplementary Materials S4**

Detailed interventions of studies included in the network meta-analysis.

| study | Drug | participant | Route | Intervention  (Experimental: control) |
| --- | --- | --- | --- | --- |
| Chen 2019 | Doxycycline | 50 | Oral | Initial dose of 200 mg 1 days, followed by 100 mg/dose per 12 h, 7days |
|  | Moxifloxacin | 50 | Intravenous | 400mg, intravenous, 1 time/d,7 days |
| Kim 2007 | Doxycycline | 45 | Oral | 200mg/day，5 days |
|  | Telithromycin | 47 | Oral | 800mg/day，5 days |
| Brown 1978 | Doxycycline | 31 | Oral | 200mg/dose，4 times/day,7 days |
|  | Tetracycline | 24 | Oral | 500mg/dose, 4 times/day, 7 days |
| Watt 2000 | Doxycycline | 28 | Oral | Initial dose of 200 mg /12h, followed by 100 mg/dose per 12 h, 7days |
|  | Rifampin | 26/24 | Oral | 300/450 mg /12h 7 days |
| Song 1995 | Doxycycline | 66 | Oral | 100 mg/dose per 12 h, 7days |
|  | Tetracycline | 50 | Oral | 500mg/dose per 6h, 7 days |
| Phimda 2007 | Azithromycin | 30 | Oral | 1g initially, followed by 500 mg once daily for 2 days |
|  | Doxycycline | 27 | Oral | 200 mg in the ﬁrst dose, followed by 100 mg every 12 h for 7 days |
| Li 2014 | Azithromycin | 23 | Intravenous | 500 mg/d, intravenous. Oral after defervescence, 500 mg/d , 3 days |
|  | Chloramphenicol | 23 | Intravenous | 1.5-2 g/d, intravenous. Dose reduced by half after, 7 days |
| Liang 2018 | Azithromycin | 55 | Intravenous | 10 mg/(kg·d), 1 time/d, 5 days |
|  | Doxycycline | 55 | Oral | 4 mg/(kg·d) (200 mg max), 2 time/d first day, follow 1 time/d, 5 days |
|  | Chloramphenicol | 55 | Intravenous | 1-2g/days, 1 time/d, 5 days |
| Ruan 2016 | Azithromycin | 30 | Intravenous | Adult: 500 mg/day. Child:10 mg/(kg·d) 5-7days |
|  | Doxycycline | 30 | Oral | Adult: 200 mg/day, 1 time/d. Child: a half of adult. 5-7days |
|  | Chloramphenicol | 30 | Intravenous | Adult: 1.5-2g/da, 1 time/d Dose reduced by defervescence. Child: 40 mg/(kg·d) 5-7days |
| Sheehy 1973 | Chloramphenicol | 30 | Oral | 3 g/day ≥ 3 days |
|  | Tetracycline | 30 | Oral | 2 g/day ≥ 3 days |
| Wei 2004 | Azithromycin | 34 | Intravenous | 10 mg/(kg·d), 1 time/d, 3 days for one course, second course after 4 days |
|  | Chloramphenicol | 33 | Intravenous | 50 mg/(kg·d), 2 time/d, Take medication until one week after the defervescence |
| Wu 2006 | Azithromycin | 52 | Intravenous | 10 mg/(kg·d), 1 time/d, 3 days for one course, second course after 4 days |
|  | Chloramphenicol | 53 | Intravenous | 50 mg/(kg·d), 2 time/d, Take medication until one week after the defervescence |
| Kim 2004 | Azithromycin | 47 | Oral | 500mg/day, 7 days |
|  | Doxycycline | 46 | Oral | 200mg/day, 7 days |
| Kim 2018 | Doxycycline | 83 | Oral | 100 mg/dose per 12 h, 5days |
|  | Rifampin | 75 | Oral | 600 mg/day，5 days |
| Zhao 2020 | Azithromycin | 75 | Intravenous | 500 mg/day, 5days |
|  | Doxycycline | 75 | Oral | Initial dose of 200 mg 1 days, followed by 100 mg/dose per day, 5 days |
| Jie 2019 | Doxycycline | 75 | Oral | Initial dose of 50 mg /12h 1day, followed by 100 mg/dose per day, 7days |
|  | Moxifloxacin | 75 | Oral | 400 mg/day，7 days |

**Supplementary Materials S5**

Specific DT outcome of studies included in the network meta-analysis.

| study | Intervene 1 | | | | Intervene 2 | | | | Intervene3 | | | |
| --- | --- | --- | --- | --- | --- | --- | --- | --- | --- | --- | --- | --- |
|  | drug 1 | Mean(h) | SD  (h) | n | drug 2 | Mean(h) | SD  (h) | n | drug 3 | Mean(h) | SD(h) | n |
| Chen 2019 | Doxycycline | 37.6 | 17.5 | 50 | moxifloxacin | 84.5 | 15.8 | 50 |  |  |  |  |
| Kim 2007 | Doxycycline | 22.6 | 21.44 | 45 | Telithromycin | 20.45 | 12.9 | 47 |  |  |  |  |
| Brown 1978 | NR |  |  |  |  |  |  |  |  |  |  |  |
| Watt 2000 | Doxycycline | 52 | 26 | 28 | Rifampin | 22.8 | 19.26 | 24 |  |  |  |  |
| Song 1995 | Doxycycline | 34 | 26.5 | 66 | Tetracycline | 37 | 26.6 | 50 |  |  |  |  |
| Phimda 2007 | Azithromycin | 64 | 35.56 | 30 | Doxycycline | 48 | 26.67 | 27 |  |  |  |  |
| Li 2014 | Azithromycin | 67.2 | 28.8 | 23 | Chloramphenicol | 69.6 | 26.4 | 23 |  |  |  |  |
| Liang 2018 | Azithromycin | 83 | 24 | 55 | Doxycycline | 82.1 | 26.2 | 55 | Chloramphenicol | 80.6 | 24 | 55 |
| Ruan 2016 | Azithromycin | 112.8 | 28.8 | 30 | Doxycycline | 50.4 | 19.2 | 30 | Chloramphenicol | 48 | 21.6 | 30 |
| Sheehy 1973 | NR |  |  |  |  |  |  |  |  |  |  |  |
| Wei 2004 | NR |  |  |  |  |  |  |  |  |  |  |  |
| Wu 2006 | Azithromycin | 56.4 | 17.28 | 52 | Chloramphenicol | 32.4 | 12.24 | 53 |  |  |  |  |
| Kim 2004 | Azithromycin | 26.33 | 16.3 | 47 | Doxycycline | 35.67 | 32.59 | 46 |  |  |  |  |
| Kim 2018 | Doxycycline | 19.33 | 8.89 | 83 | Rifampin | 17.33 | 10.37 | 75 |  |  |  |  |
| Zhao2020 | Azithromycin | 83.28 | 26.88 | 75 | Doxycycline | 82.32 | 26.64 | 75 |  |  |  |  |
| Jie 2019 | Doxycycline | 54.48 | 22.8 | 75 | moxifloxacin | 62.16 | 23.52 | 75 |  |  |  |  |

**Supplementary Materials S6**

Conversion formula:

If the study is missing the mean or/and standard deviation, use quartiles, medians and extremes to obtain an approximation.


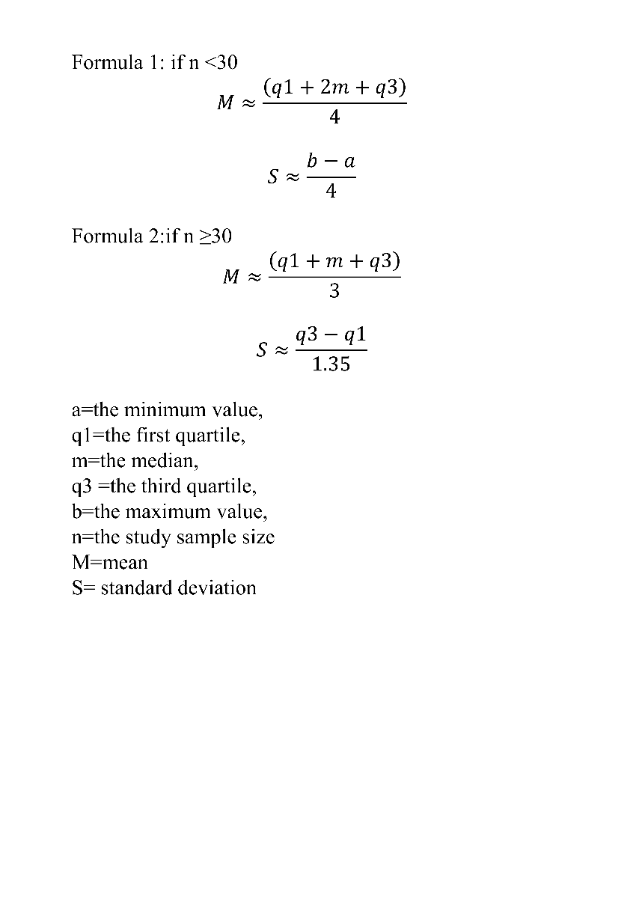


**Supplementary Materials S7**

Risk of bias analysis detailed for each single study included in the NMA.


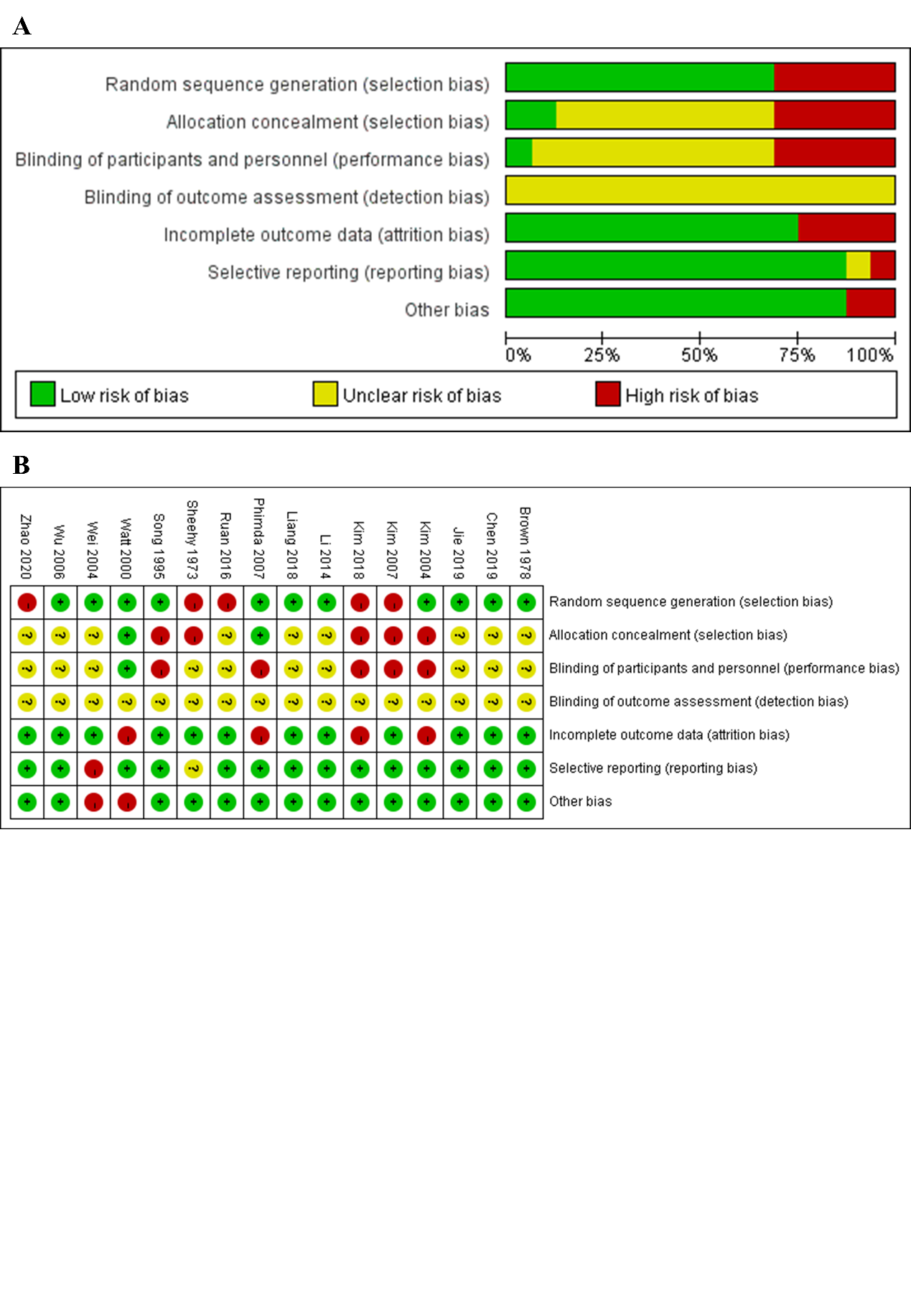


**Supplementary Materials S8**

Bayesian ranking results

The number in each cell represents the posterior probability of the row-defining treatment being ranked at the column-defining position. The numbers with biggest probability of ranking first are in bold.

**CR group**

|  | rank1 | rank 2 | rank 3 | rank 4 | rank 5 | rank 6 | rank 7 | SURCA |
| --- | --- | --- | --- | --- | --- | --- | --- | --- |
| Azithromycin | 0.051 | 0.179 | 0.264 | 0.255 | 0.173 | 0.077 | 0.002 | 57% |
| Chloramphenicol | 0.118 | 0.269 | 0.268 | 0.174 | 0.113 | 0.057 | 0.002 | 65% |
| Doxycycline | 0.004 | 0.041 | 0.114 | 0.240 | 0.374 | 0.226 | 0.001 | 40% |
| Moxifloxacin | 0.000 | 0.001 | 0.001 | 0.002 | 0.007 | 0.129 | 0.859 | 3% |
| **Rifampin** | **0.536** | 0.198 | 0.092 | 0.071 | 0.057 | 0.042 | 0.003 | 82% |
| Telithromycin | 0.212 | 0.140 | 0.072 | 0.069 | 0.084 | 0.298 | 0.125 | 50% |
| Tetracycline | 0.079 | 0.172 | 0.188 | 0.189 | 0.193 | 0.170 | 0.008 | 54% |

**DT group**

|  | rank 1 | rank 2 | rank 3 | rank 4 | rank 5 | rank 6 | rank 7 | SURCA |
| --- | --- | --- | --- | --- | --- | --- | --- | --- |
| Azithromycin | 0.129 | 0.339 | 0.285 | 0.146 | 0.069 | 0.027 | 0.004 | 70% |
| Chloramphenicol | 0.014 | 0.050 | 0.121 | 0.166 | 0.218 | 0.261 | 0.169 | 34% |
| Doxycycline | 0.003 | 0.036 | 0.178 | 0.3427 | 0.306 | 0.119 | 0.017 | 44.3% |
| Moxifloxacin | 0.555 | 0.219 | 0.1 | 0.055 | 0.035 | 0.024 | 0.011 | 85% |
| **Rifampin** | **0.019** | 0.046 | 0.066 | 0.083 | 0.134 | 0.25 | 0.401 | 23% |
| Telithromycin | 0.119 | 0.141 | 0.119 | 0.105 | 0.121 | 0.169 | 0.225 | 43.7% |
| Tetracycline | 0.159 | 0.167 | 0.131 | 0.103 | 0.117 | 0.149 | 0.173 | 50% |

**GS-AE group**

|  | rank 1 | rank 2 | rank 3 | rank 4 | rank 5 | rank 6 | rank 7 | SURCA |
| --- | --- | --- | --- | --- | --- | --- | --- | --- |
| Azithromycin | 0.024 | 0.053 | 0.099 | 0.184 | 0.263 | 0.254 | 0.122 | 36% |
| **Chloramphenicol** | **0.031** | 0.051 | 0.08 | 0.140 | 0.213 | 0.261 | 0.225 | 31% |
| Doxycycline | 0.124 | 0.343 | 0.329 | 0.153 | 0.043 | 0.007 | 0.001 | 72% |
| Moxifloxacin | 0.107 | 0.109 | 0.123 | 0.158 | 0.156 | 0.169 | 0.178 | 44% |
| Rifampin | 0.256 | 0.174 | 0.147 | 0.138 | 0.113 | 0.097 | 0.075 | 62% |
| Telithromycin | 0.149 | 0.092 | 0.084 | 0.103 | 0.113 | 0.127 | 0.331 | 39% |
| Tetracycline | 0.309 | 0.179 | 0.136 | 0.123 | 0.099 | 0.084 | 0.068 | 66% |

**ABC-AE group**

|  | rank 1 | rank 2 | rank 3 | SURCA |
| --- | --- | --- | --- | --- |
| **Azithromycin** | **0.002** | 0.036 | 0.961 | 2% |
| Chloramphenicol | 0.316 | 0.658 | 0.026 | 65% |
| Doxycycline | 0.682 | 0.306 | 0.012 | 83% |

**Supplementary Materials S9**

Inconsistency of the network meta-analysis.


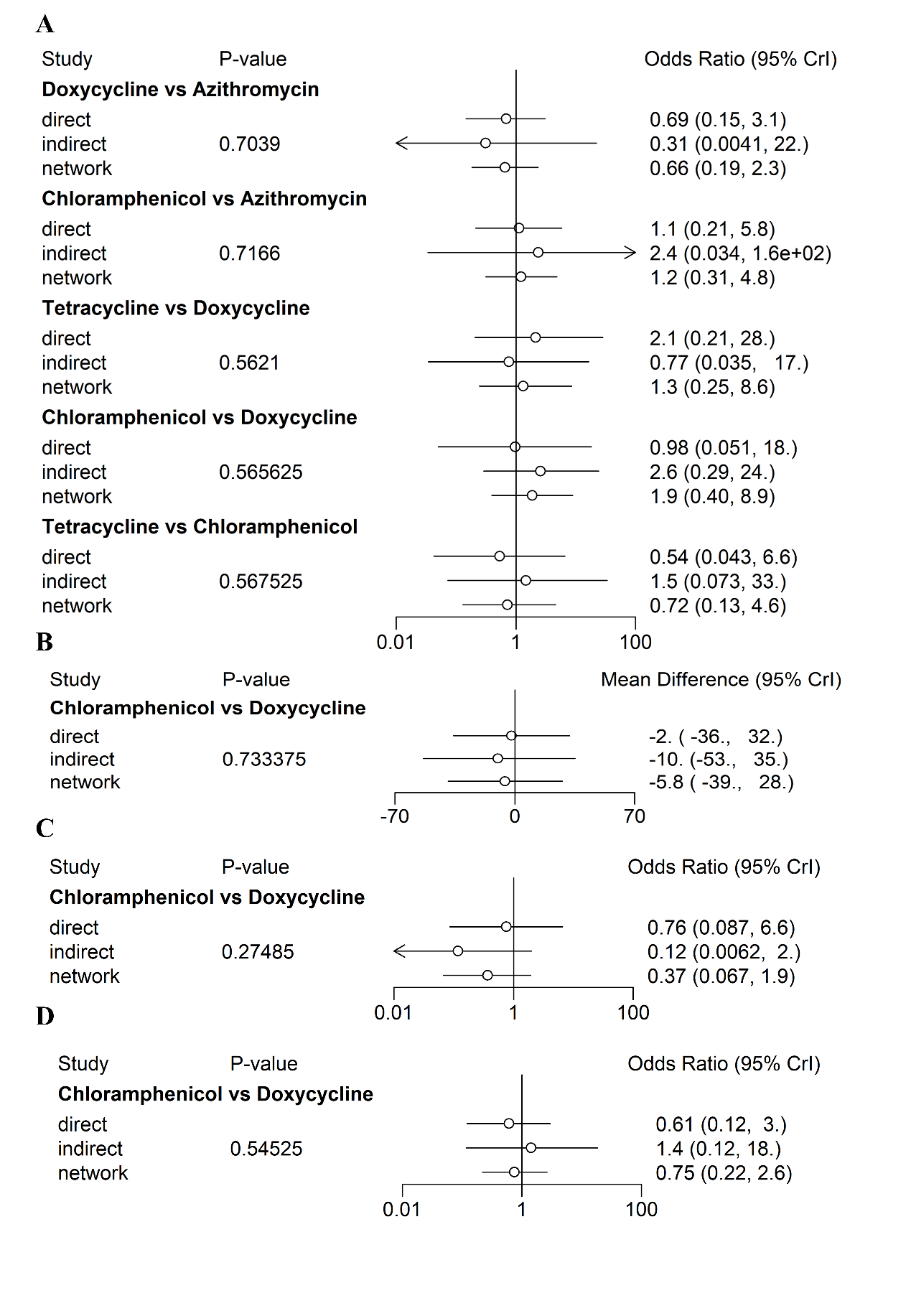


**Supplementary Materials S10**

Heterogeneity of the network meta-analysis.


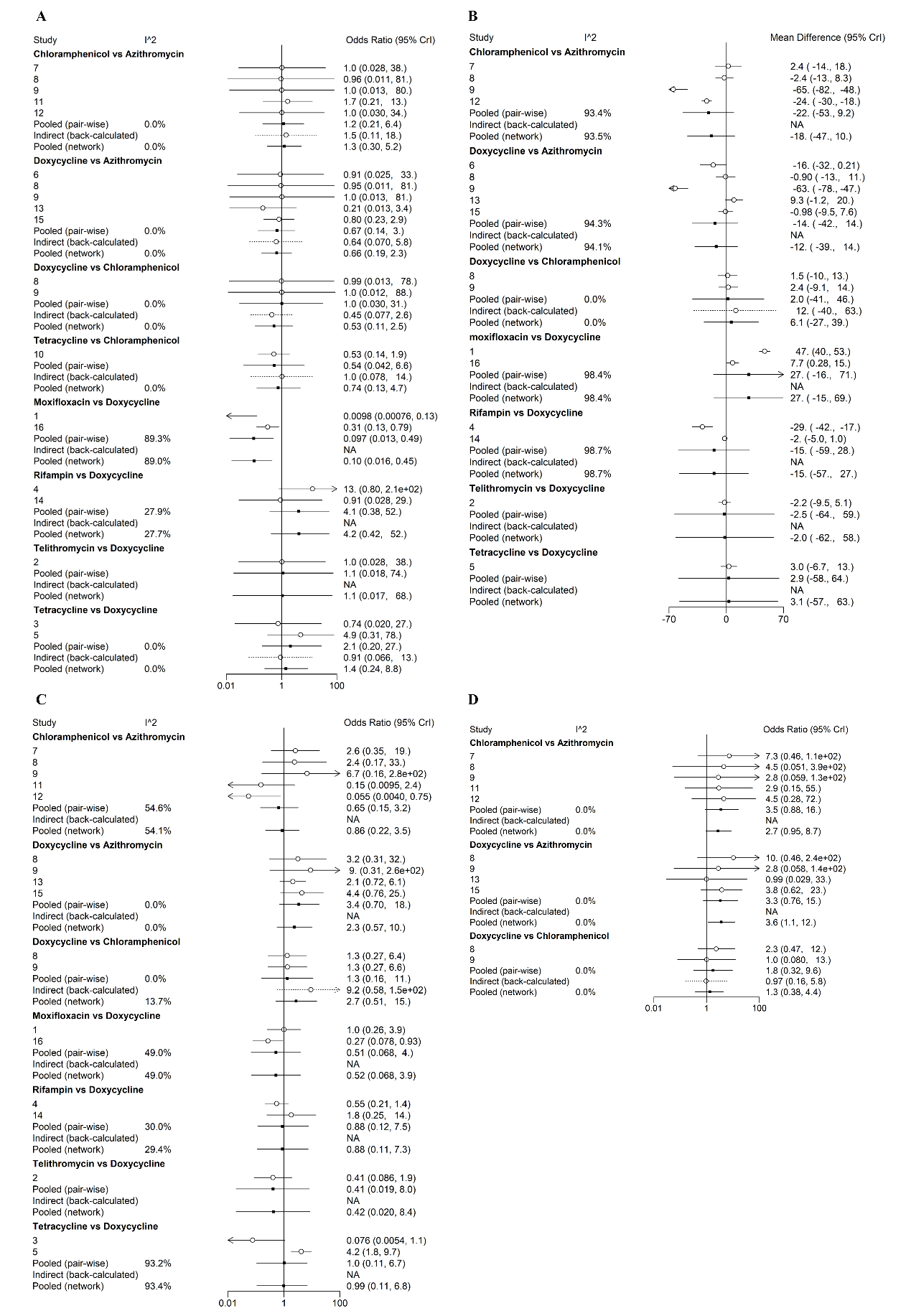


**Supplementary Materials S11**


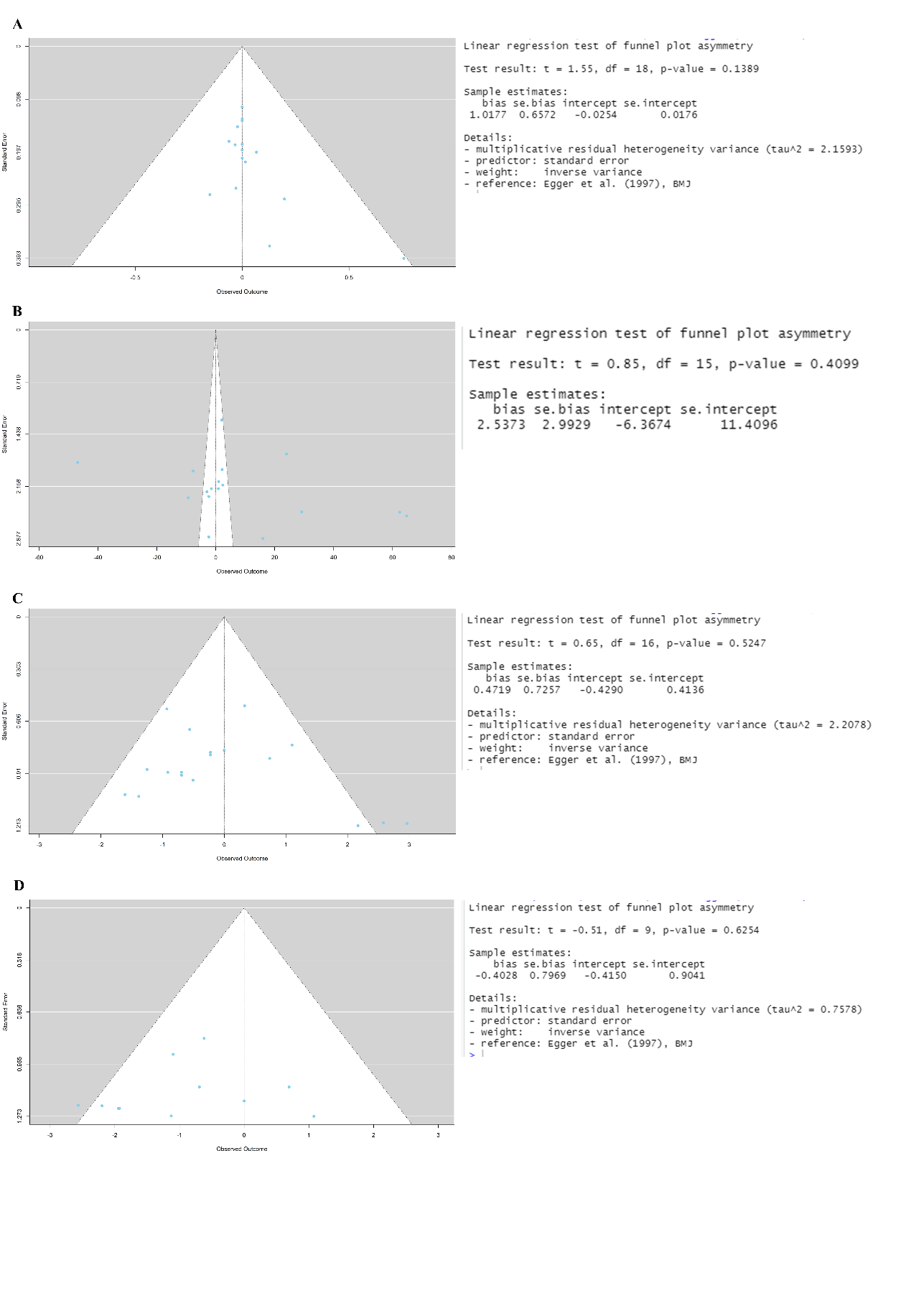


**Supplementary Materials S12**

Brooks-Gelman-Rubin history and diagnostic of the network meta-analysis.


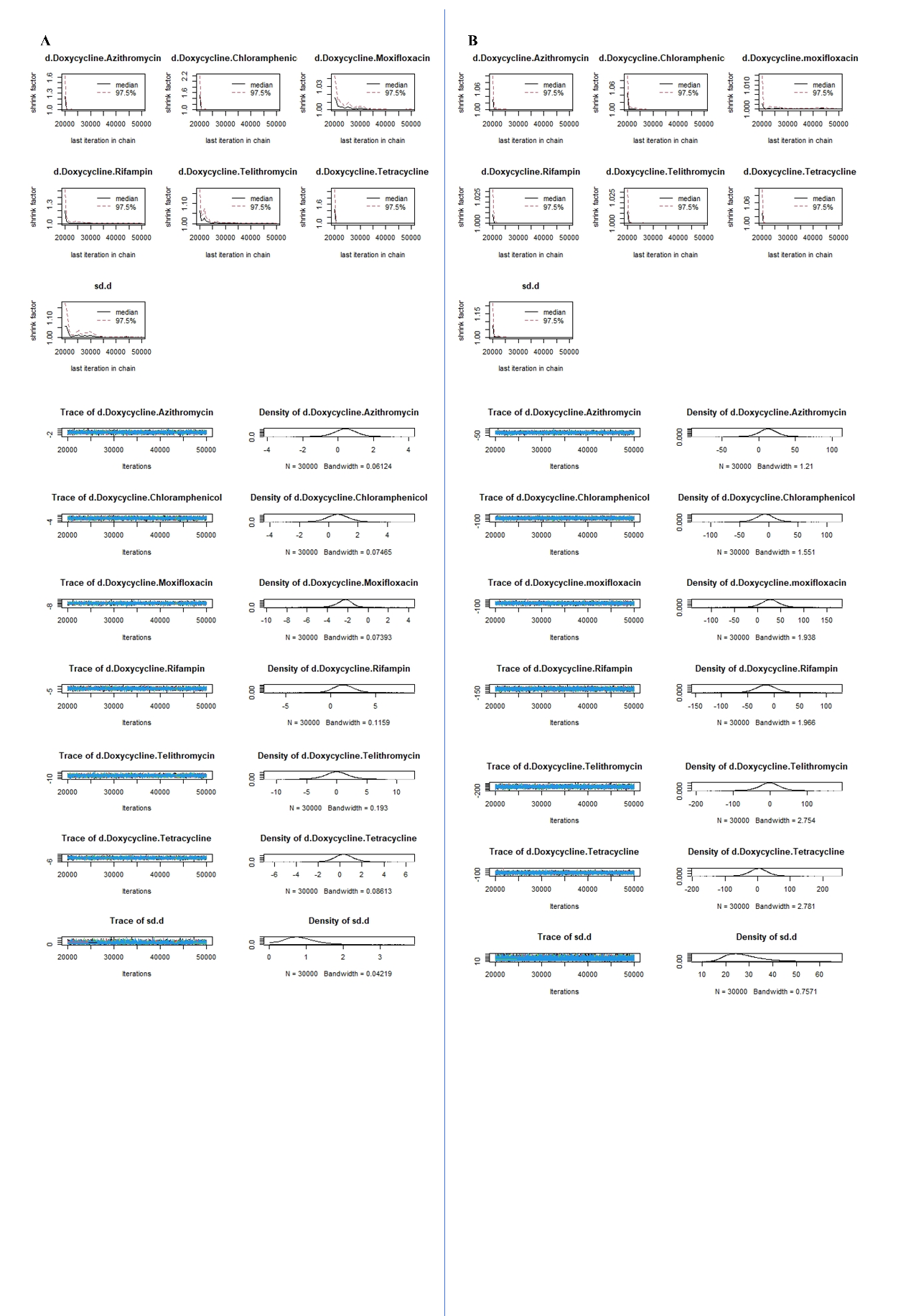


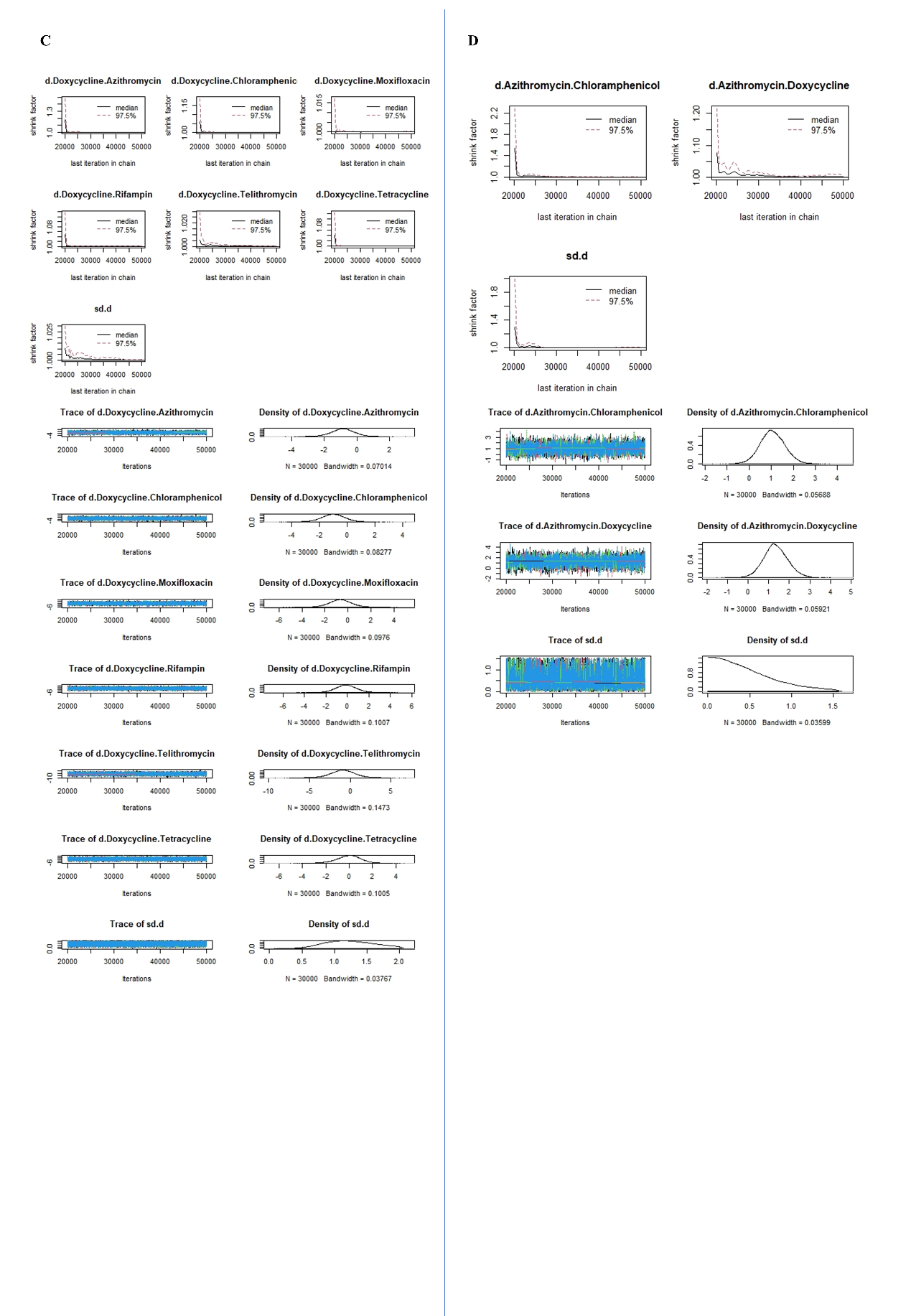


**Supplementary Materials S13**

**GRADE ratings for CR**

| **Comparsion** | **No of studies** | **Within-study bias** | **Imprecision** | **Heterogeneity** | **Incoherence** | **Indirect-ness** | **Across-studies bias** | **Confidence rating** |
| --- | --- | --- | --- | --- | --- | --- | --- | --- |
| Azithromycin:  Doxycycline | 5 | Major concerns | No concerns | No  concerns | No  concerns | No concerns | Undetected | Moderate |
| Azithromycin:  Chloramphenicol | 5 | Major concerns | No concerns | No  concerns | No  concerns | No concerns | Undetected | Moderate |
| Azithromycin:  Tetracycline | 0 | Major concerns | Major concerns | No  concerns | No concerns | No concerns | Undetected | Very Low |
| Azithromycin:  Rifampin | 0 | Major concerns | Major concerns | No  concerns | No concerns | No concerns | Undetected | Very Low |
| Azithromycin:  Telithromycin | 0 | Major concerns | Major concerns | No  concerns | No concerns | No concerns | Undetected | Very Low |
| Azithromycin:  Moxifloxacin | 0 | Major concerns | Major concerns | No  concerns | No concerns | No concerns | Undetected | Very Low |
| Doxycycline:  Chloramphenicol | 2 | Major concerns | No concerns | No  concerns | No concerns | No concerns | Undetected | Moderate |
| Doxycycline:  Tetracycline | 2 | Major concerns | No concerns | No  concerns | No concerns | No concerns | Undetected | Moderate |
| Doxycycline:  Rifampin | 2 | Major concerns | No concerns | Some concerns | No concerns | No concerns | Undetected | Low |
| Doxycycline:  Telithromycin | 1 | Major concerns | Major concerns | No  concerns | No concerns | No concerns | Undetected | Very Low |
| Doxycycline:  Moxifloxacin | 2 | Major concerns | Some concerns | Major concerns | No concerns | No concerns | Undetected | Very Low |
| Chloramphenicol:  Tetracycline | 1 | Major concerns | Major concerns | No  concerns | No concerns | No concerns | Undetected | Very Low |
| Chloramphenicol:  Rifampin | 0 | Major concerns | Major concerns | No  concerns | No concerns | No concerns | Undetected | Very Low |
| Chloramphenicol:  Telithromycin | 0 | Major concerns | Major concerns | No  concerns | No concerns | No concerns | Undetected | Very Low |
| Chloramphenicol:  Moxifloxacin | 0 | Major concerns | Major concerns | No  concerns | No concerns | No concerns | Undetected | Very Low |
| Tetracycline:  Rifampin | 0 | Major concerns | Major concerns | No  concerns | No concerns | No concerns | Undetected | Very Low |
| Tetracycline:  Telithromycin | 0 | Major concerns | Major concerns | No  concerns | No concerns | No concerns | Undetected | Very Low |
| Tetracycline:  Moxifloxacin | 0 | Major concerns | Major concerns | No  concerns | No concerns | No concerns | Undetected | Very Low |
| Rifampin:  Telithromycin | 0 | Major concerns | Major concerns | No  concerns | No concerns | No concerns | Undetected | Very Low |
| Rifampin:  Moxifloxacin | 0 | Major concerns | Major concerns | No  concerns | No concerns | No concerns | Undetected | Very Low |
| Telithromycin:  Moxifloxacin | 0 | Major concerns | Major concerns | No  concerns | No concerns | No concerns | Undetected | Very Low |

**GRADE ratings for GS-AD**

| **Comparsion** | **No of studies** | **Within-study bias** | **Imprecision** | **Heterogeneity** | **Incoherence** | **Indirect-ness** | **Across-studies bias** | **Confidence rating** |
| --- | --- | --- | --- | --- | --- | --- | --- | --- |
| Azithromycin:  Doxycycline | 4 | Major concerns | Major concerns | No  concerns | No concerns | No concerns | Undetected | Very Low |
| Azithromycin:  Chloramphenicol | 5 | Major concerns | No concerns | Major concerns | No concerns | No concerns | Undetected | Very Low |
| Azithromycin:  Tetracycline | 0 | Major concerns | Major concerns | No  concerns | No concerns | No concerns | Undetected | Very Low |
| Azithromycin:  Rifampin | 0 | Major concerns | Major concerns | No  concerns | No concerns | No concerns | Undetected | Very Low |
| Azithromycin:  Telithromycin | 0 | Major concerns | Major concerns | No  concerns | No concerns | No concerns | Undetected | Very Low |
| Azithromycin:  Moxifloxacin | 0 | Major concerns | Major concerns | No  concerns | No concerns | No concerns | Undetected | Very Low |
| Doxycycline:  Chloramphenicol | 2 | Major concerns | Major concerns | No  concerns | No concerns | No concerns | Undetected | Very Low |
| Doxycycline:  Tetracycline | 2 | Major concerns | Some concerns | Major concerns | No concerns | No concerns | Undetected | Very Low |
| Doxycycline:  Rifampin | 2 | Major concerns | Major concerns | Some concerns | No concerns | No concerns | Undetected | Very Low |
| Doxycycline:  Telithromycin | 1 | Major concerns | Major concerns | No  concerns | No concerns | No concerns | Undetected | Very Low |
| Doxycycline:  Moxifloxacin | 2 | Major concerns | Major concerns | Some concerns | No concerns | No concerns | Undetected | Very Low |
| Chloramphenicol:  Tetracycline | 0 | Major concerns | Major concerns | No  concerns | No concerns | No concerns | Undetected | Very Low |
| Chloramphenicol:  Rifampin | 0 | Major concerns | Major concerns | No  concerns | No concerns | No concerns | Undetected | Very Low |
| Chloramphenicol:  Telithromycin | 0 | Major concerns | Major concerns | No  concerns | No concerns | No concerns | Undetected | Very Low |
| Chloramphenicol:  Moxifloxacin | 0 | Major concerns | Major concerns | No  concerns | No concerns | No concerns | Undetected | Very Low |
| Tetracycline:  Rifampin | 0 | Major concerns | Major concerns | No  concerns | No concerns | No concerns | Undetected | Very Low |
| Tetracycline:  Telithromycin | 0 | Major concerns | Major concerns | No  concerns | No concerns | No concerns | Undetected | Very Low |
| Tetracycline:  Moxifloxacin | 0 | Major concerns | Major concerns | No  concerns | No concerns | No concerns | Undetected | Very Low |
| Rifampin:  Telithromycin | 0 | Major concerns | Major concerns | No  concerns | No concerns | No concerns | Undetected | Very Low |
| Rifampin:  Moxifloxacin | 0 | Major concerns | Major concerns | No  concerns | No concerns | No concerns | Undetected | Very Low |
| Telithromycin:  Moxifloxacin | 0 | Major concerns | Major concerns | No  concerns | No concerns | No concerns | Undetected | Very Low |

**GRADE ratings for DT**

| **Comparsion** | **No of studies** | **Within-study bias** | **Imprecision** | **Heterogeneity** | **Incoherence** | **Indirect-ness** | **Across-studies bias** | **Confidence rating** |
| --- | --- | --- | --- | --- | --- | --- | --- | --- |
| Azithromycin:  Doxycycline | 5 | Major concerns | Major concerns | Major concerns | No concerns | No concerns | Undetected | Very Low |
| Azithromycin:  Chloramphenicol | 4 | Major concerns | Major concerns | Major concerns | No concerns | No concerns | Undetected | Very Low |
| Azithromycin:  Tetracycline | 0 | Major concerns | Major concerns | No  concerns | No concerns | No concerns | Undetected | Very Low |
| Azithromycin:  Rifampin | 0 | Major concerns | Major concerns | No  concerns | No concerns | No concerns | Undetected | Very Low |
| Azithromycin:  Telithromycin | 0 | Major concerns | Major concerns | No  concerns | No concerns | No concerns | Undetected | Very Low |
| Azithromycin:  Moxifloxacin | 0 | Major concerns | Major concerns | No  concerns | No concerns | No concerns | Undetected | Very Low |
| Doxycycline:  Chloramphenicol | 2 | Major concerns | Major concerns | No  concerns | No concerns | No concerns | Undetected | Very Low |
| Doxycycline:  Tetracycline | 1 | Major concerns | Major concerns | No  concerns | No concerns | No concerns | Undetected | Very Low |
| Doxycycline:  Rifampin | 2 | Major concerns | Major concerns | Major concerns | No concerns | No concerns | Undetected | Very Low |
| Doxycycline:  Telithromycin | 1 | Major concerns | Major concerns | No  concerns | No concerns | No concerns | Undetected | Very Low |
| Doxycycline:  Moxifloxacin | 2 | Major concerns | Major concerns | Major concerns | No concerns | No concerns | Undetected | Very Low |
| Chloramphenicol:  Tetracycline | 0 | Major concerns | Major concerns | No  concerns | No concerns | No concerns | Undetected | Very Low |
| Chloramphenicol:  Rifampin | 0 | Major concerns | Major concerns | No  concerns | No concerns | No concerns | Undetected | Very Low |
| Chloramphenicol:  Telithromycin | 0 | Major concerns | Major concerns | No  concerns | No concerns | No concerns | Undetected | Very Low |
| Chloramphenicol:  Moxifloxacin | 0 | Major concerns | Major concerns | No  concerns | No concerns | No concerns | Undetected | Very Low |
| Tetracycline:  Rifampin | 0 | Major concerns | Major concerns | No  concerns | No concerns | No concerns | Undetected | Very Low |
| Tetracycline:  Telithromycin | 0 | Major concerns | Major concerns | No  concerns | No concerns | No concerns | Undetected | Very Low |
| Tetracycline:  Moxifloxacin | 0 | Major concerns | Major concerns | No  concerns | No concerns | No concerns | Undetected | Very Low |
| Rifampin:  Telithromycin | 0 | Major concerns | Major concerns | No  concerns | No concerns | No concerns | Undetected | Very Low |
| Rifampin:  Moxifloxacin | 0 | Major concerns | Major concerns | No  concerns | No concerns | No concerns | Undetected | Very Low |
| Telithromycin:  Moxifloxacin | 0 | Major concerns | Major concerns | No  concerns | No concerns | No concerns | Undetected | Very Low |

**GRADE ratings for ABC-AD**

| **Comparsion** | **No of studies** | **Within-study bias** | **Imprecision** | **Heterogeneity** | **Incoherence** | **Indirect-ness** | **Across-studies bias** | **Confidence rating** |
| --- | --- | --- | --- | --- | --- | --- | --- | --- |
| Azithromycin:  Doxycycline | 4 | Major concerns | No concerns | No  concerns | No concerns | No concerns | Undetected | Moderate |
| Azithromycin:  Chloramphenicol | 5 | Major concerns | No concerns | No  concerns | No concerns | No concerns | Undetected | Moderate |
| Doxycycline:  Chloramphenicol | 2 | Major concerns | No concerns | No  concerns | No concerns | No concerns | Undetected | Moderate |

**Within-study bias**: All included studies were at high risk of bias for detection bias,15/16 included studies were at high risk of bias for performance bias and 14/16 included studies were at high risk of bias for selection bias We therefore judged that there were ‘Major concern’ for all the NMA estimates.

**Imprecision**: To assess imprecision of the estimates, we set the threshold for the smallest worthwhile difference at 1 point in each term. A rating of ‘major concerns’ is assigned to a treatment effect if the 95% confidence interval extends beyond the area of equivalence on the opposite side of the no effect line as the point estimate, so that the estimated treatment effect is compatible with clinically important effects in both directions. A rating of ‘some concerns’ is assigned if the confidence interval extends into but not beyond the area of equivalence on the opposite side of the no effect line. There are ‘No concerns’ if the confidence interval is entirely on one side of the no effect line, or if it is entirely within the area of equivalence. If there are 0 studies in a term, it is rated as a ‘Major concern’.

**Heterogeneity**: Heterogeneity was considered ‘No concerns’, ‘Some concerns’, ‘major concerns’ for estimated I^2^ under 25%, between 25% and 50%, and over 50% in each term.

**Incoherence**: Incoherence was assessed by the global test of inconsistency (design-by-treatment test) and the local tests of inconsistency (back-calculation), none of which were statistically significant for all three NMAs.

**Indirectness**: We judged that there was no indirectness because the included studies all fit our research questions.

**Across-studies bias**: There was no evidence of publication bias, as the funnel plot did not suggest the absence of any bias (p>0.05). The included studies had few missing outcomes and we judged the potential for reporting bias was low. Therefore, all trials were at low risk of Across-studies bias .

**Supplementary Materials S14**

Sensitivity analysis of the network meta-analysis.

The sensitivity for 3 outcomes (Sensitivity analysis and regression analysis were not performed because of the small number of studies in the ADB group) were evaluated by repeating network meta analyses following sequentially exclusion of studies with 5 studies that did not use randomized subgroups (A), 3 studies with publications prior to 2000(B), 2 studies of scrub typhus in adolescents(C), 3 studies with one group of less than 30 patients(D), and 7 studies that included intravenous drug administration(E). After exclusion of these trials the hierarchy did not change considerably in CR, ADG and DT.

Tests of heterogeneity was as follows: In ADG group, there was a significant reduction in pair-meta heterogeneity (I^2^ pair) and network heterogeneity (I^2^ network) of the study when publications prior to 2000 studies or less than 30 patients studies were excluded. In groups CR and DT, sensitivity analysis did not reveal a noticeable change in heterogeneity.

**a.**


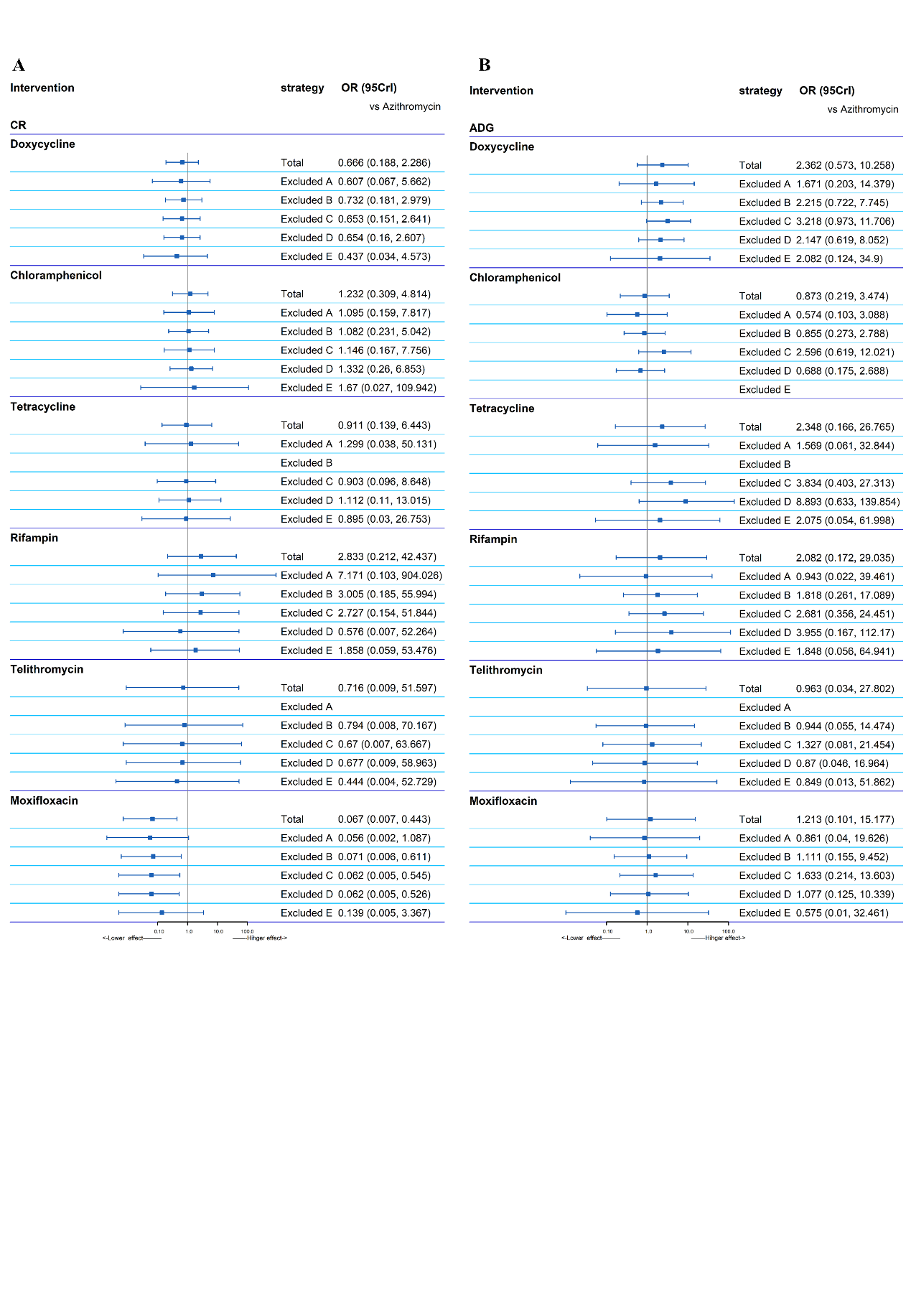


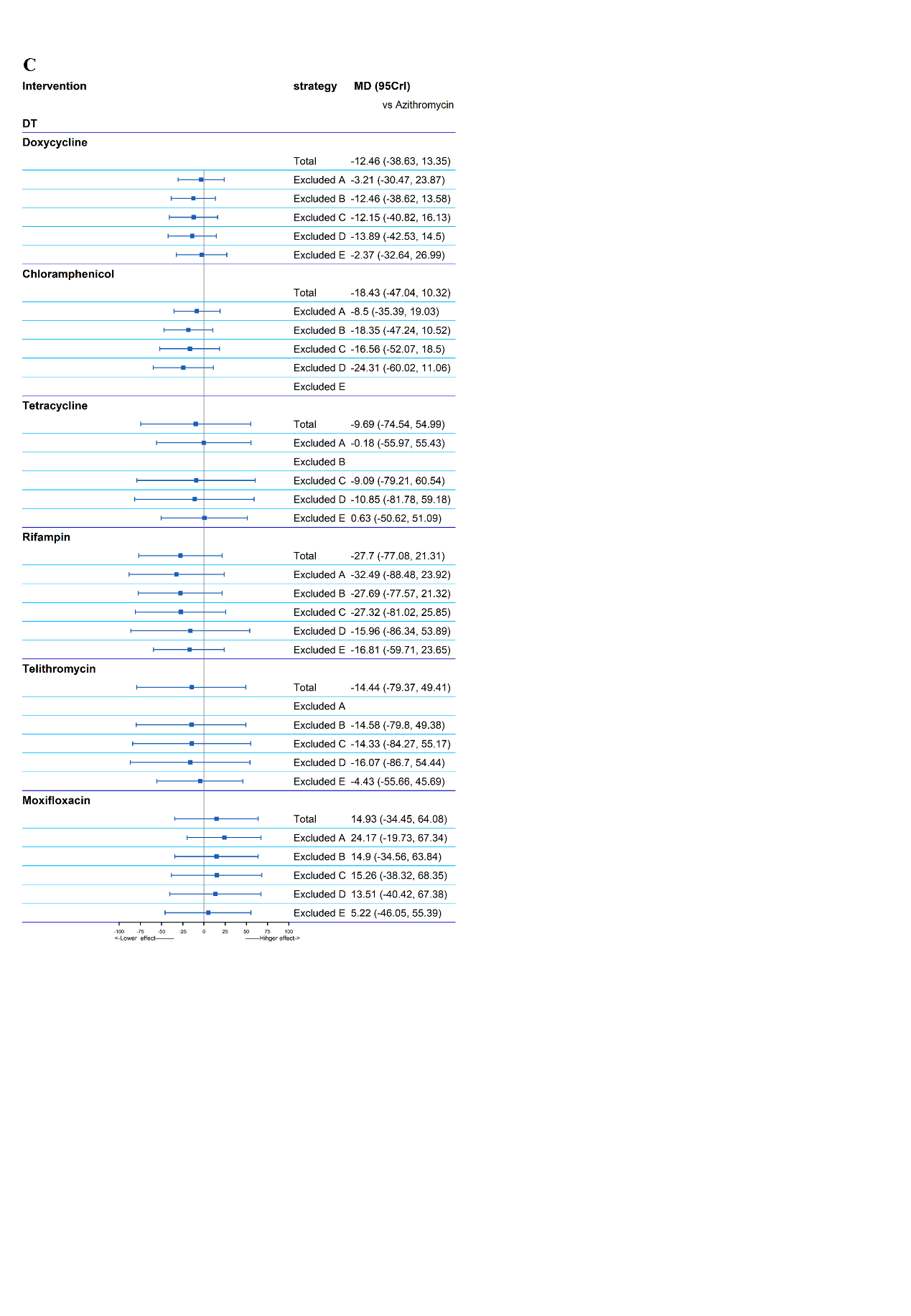


**b.**

Heterogeneity test for each group during sensitivity analysis

|  | CR | | GS-AD | | DT | |
| --- | --- | --- | --- | --- | --- | --- |
| CR | I^2^ pair (%) | I^2^ network | I^2^ pair (%) | I^2^ network | I^2^ pair (%) | I^2^ network |
| total | 2.81 | 0 | 60.18 | 59.41 | 96.06 | 95.56 |
| Excluded A | 36.51 | 28.61 | 76.17 | 73.6 | 94.96 | 94.05 |
| Excluded B | 5.39 | 0 | 23.16 | 24.39 | 96.06 | 95.66 |
| Excluded C | 19.06 | 7.32 | 49.14 | 42.39 | 96.45 | 95.96 |
| Excluded D | 13.82 | 2.58 | 14.82 | 28.6 | 95.45 | 94.79 |
| Excluded E | 0 | 0 | 88.03 | 87.93 | 97.42 | 97.42 |

**Supplementary Materials S15**

meta regressions analysis of the network meta-analysis.

| CR (k=16) | Coefficient  (95% confidence intervals) | z | P value |
| --- | --- | --- | --- |
| Study blinding | 0.001 (-0.2 to 0.201) | 0.010 | 0.992 |
| Male rate | -0.058(0.996 to 0.879) | -0.122 | 0.903 |
| Mean age | -0.0004 (-0.007 to 0.007) | -0.115 | 0.908 |
| Publication year | -0.001 (-0.008 to 0.006) | -0.317 | 0.752 |
|  |  |  |  |
| GS-AD(k=14) | Coefficient  (95% confidence intervals) | z | P value |
| Study blinding | -0.23 (-1.339 to 0.878) | 0.566 | 0.684 |
| Male rate | 0.42 (0.004 to 0.081) | 2.132 | 0.033 |
| Mean age | 0.03 (0.003 to 0.057) | 2.168 | 0.030 |
| Publication year | 0.015 (-0.032 to 0.062) | 0.024 | 0.524 |
|  |  |  |  |
| DT(k=13) | Coefficient  (95% confidence intervals) | z | P value |
| Study blinding | 8.71 (4.80 to 12.62) | 4.368 | <0.001 |
| Male rate | 2.39 (-12.95 to 17.74) | 0.306 | 0.759 |
| Mean age | -0.07 (-0.18 to 0.03) | -1.324 | 0.185 |
| Publication year | -0.34 (-0.62 to -0.06) | -2.35 | 0.018 |
